# Risk attitudes and human mobility during the COVID-19 pandemic

**DOI:** 10.1038/s41598-020-76763-2

**Published:** 2020-11-16

**Authors:** Ho Fai Chan, Ahmed Skali, David A. Savage, David Stadelmann, Benno Torgler

**Affiliations:** 1 School of Economics and Finance, Queensland University of Technology, Brisbane, Australia; 2 Centre for Behavioural Economics, Society and Technology (BEST), Brisbane, Australia; 3 Department of Economics, Deakin University, Burwood, Australia; 4 Newcastle Business School, University of Newcastle, Callaghan, Australia; 5 University of Bayreuth, Bayreuth, Germany; 6 IREF—Institute for Research in Economic and Fiscal Issues, Paris, France; 7 CREMA—Center for Research in Economics, Management, and the Arts, Basel, Switzerland

**Keywords:** Psychology, Human behaviour, Public health

## Abstract

Behavioural responses to pandemics are less shaped by actual mortality or hospitalisation risks than they are by risk attitudes. We explore human mobility patterns as a measure of behavioural responses during the COVID-19 pandemic. Our results indicate that risk-taking attitudes are a critical factor in predicting reductions in human mobility and social confinement around the globe. We find that the sharp decline in mobility after the WHO (World Health Organization) declared COVID-19 to be a pandemic can be attributed to risk attitudes. Our results suggest that regions with risk-averse attitudes are more likely to adjust their behavioural activity in response to the declaration of a pandemic even before official government lockdowns. Further understanding of the basis of responses to epidemics, e.g., precautionary behaviour, will help improve the containment of the spread of the virus.

## Introduction

*In Thackeray’s novel Henry Esmond, for instance, this dread informs the narrative. The heroine, Lady Castlewood, contracts the disease as an adult. Her husband had been a brave soldier in combat, but he was unable to face a malady that he could not fight and that threatened him not only with death, but also with disfigurement. Unwilling to put his pink complexion and his fair hair at risk, Lord Castlewood took to his heels and deserted his household for the duration of the outbreak. But he was not part of a mass exodus, even though Henry Esmond declares that smallpox was “that dreadful scourge of the world” and a “withering blight” and “pestilence” that “would enter a village and destroy half its inhabitants.” * Snowden (2019, p. 101).

The central features of modern global society, i.e., globalisation and urbanisation, make us more vulnerable to the challenge of pandemic diseases; and their global implications can trigger large-scale responses^1^. Epidemics such as COVID-19 are a threat to our social fabric^2^, thus it is important to understand such occurrences from the perspective of risk attitudes. Scholars have emphasised that social and behavioural sciences can offer important insights into how the COVID-19 pandemic may be better understood and managed^3–6^. Until recently, risky behaviour has been predominantly analysed in relation to the HIV/AIDS pandemic^7,8^. However, studies have also tried to model the effects of risk perceptions on the spread of epidemics^9^, and have explored how different levels of awareness may help to prevent an outbreak^10^ or limit the replication rates. Other studies have explored the implications of risk attitudes in disasters^11–14^ or extreme situations^15^.

Here, we take a look at how risk attitudes affect human mobility during the COVID-19 pandemic. To measure risk-taking attitudes, we use the Global Preferences Survey^16,17^, which assess individual’s risk preference by combining experimental lottery choice sequences using a staircase method and self-assessment based on the willingness to take risks (see “Methods” section for details). We then geocode this data to obtain measure aggregates at the regional level. Exploring how risk attitudes affect social mobility at the regional level is interesting as risk behaviour can be seen as the product of an interplay between individuals, actions of others, and the community or social environment^7^. Risk is deeply embedded in specific socio-cultural backgrounds^18^, with country and geographical differences in risk-taking reported by scholars such as^16^ (e.g., higher risk-taking values in Africa and the Middle East, while Western European countries are relatively risk-averse). In the context of a pandemic where a population is attempting social isolation or lockdown, we observe that shopping behaviours change (drop) and large swathes of the workforce have lost their jobs, which means that the entire population has been directly affected by the consequences of the pandemic, if not by the health consequences of the virus. Therefore, it is essential to explore how citizens’ responses to an epidemic are driven by risk attitudes or preferences at the community or regional level. We also explore how individual behaviour responses to global announcements – such as the COVID-19 outbreak classification as a pandemic by the WHO – can be shaped by risk attitudes.

## Background

Risk-taking attitudes and behaviours are important elements of human behaviour as they determine a range of decision-making strategies^19^ and contribute to people’s ability to navigate a complex, uncertain, and dangerous world, where risk looms large^13^. Research has shown that risk aversion can result in the over-weighting of risk factors while risk-seeking can result in the under-weighting of risk^20–24^. Advanced civilisations dating back to the Asipu in Mesopotamia in 3200 B.C. had risk management strategies in place to estimate profits/losses or successes/failures (^25^ discussed in^26^). Another early example of risk analysis and risk management can be found in the *Code of Hammurabi* issued in 1950 B.C.^26^. Our evolution has equipped us with a cognitive apparatus enabling us to monitor danger during our daily activities^27^, as these enduring and recurring risks in our environment have required evolutionary adaptiveness as a core selective factor for survival^28^. The implication is that we must remain safe to guarantee our survival as such it comes as no surprise that we are all innately aware of the proverb “Better safe than sorry”.

Risk entails a probabilistic assessment of something occurring, which then allows for a decision regarding what action to take. However, not only are we boundedly rational human beings^29^ subject to emotions^30^ such as fear, but also to the complexity of the environment and situation^13^. Often the limited available information on contextual factors of other humans, or dynamic changes, may not allow us to have a clear idea about the actual probabilities we face in uncommon situations such as a pandemic. In addition, calculating the probability of risk is not the same as actually perceiving it, and humans use quick but often less accurate heuristics to make judgments that also include perceptions of risk. Our biases may disrupt our risk assessments in both positive and negative ways by limiting access to information (searches), limited cognitive understanding (noise), and through our personal experiences. Thus, subjective perceptions or emotional responses may be triggered by human traits or other factors. For example, we judge risk differently based on the physical distance between ourselves and a given source of danger, i.e., we feel safer if the danger is further away, and we are less likely to monitor it over an extended duration^31^. This may work relatively well for traditional dangers like fires or floods, but the spread of a pandemic is invisible, and only media reports or those in hospital provide any clues to its presence. As such, we likely fail to correctly use local transmission (infection) rates as a guide of the pandemic’s proximity or distance to us, and the level of threat it poses. Risk as a feeling is less driven by actual probabilities and more by our instinctive and intuitive reaction to danger [^27^, p. 70]. Risk-taking has often been classified as a stable personality trait^32^, although situational or contextual factors can also matter (see, e.g.,^33–35^). An individual’s risk preferences and their perception of risk are highly correlated, such that they interact to exacerbate the underlying risk type. In other words, risk seekers are likely to have less precise perceptions of risk; not only are they willing to accept more gambles, but their estimations of the risks associated with the gambles are underweighted, leading to greater adoption of risk than the individual intended^36^. In addition, humans are also subject to framing biases, reacting differently depending on how information is presented (e.g., positively or negatively, see^37^). This framing can increase or decrease our willingness to take or avoid risk, especially where losses are concerned—the loss of life from contracting the virus is the ultimate loss. Thus, while generally considered stable, preferences are not set in stone and are open to change, especially after we experience losses; i.e., an individual may be more risk-seeking following losses and more risk-averse following gains^14,38–40^.

Feelings elicited during a pandemic have an impact on everyday activities^41^ and individuals are required to make trade-offs that are affected by their risk behaviour. Is it safe to go out shopping, to the park, to use public transportation, and so on? What are the chances of getting infected? How do we need to respond? Risk attitudes matter as individuals are aware that going into public places increases the possibility of being infected; if there were to be an infection, this would be subsequently regretted. Risk-averse individuals may respond more to unfamiliar risks that are perceived as uncontrollable^42^. During pandemics, states may also become more controlling—historically, social mobility restrictions or regulations are common in pandemics. For example, in the Middle Ages, anti-plague regulations banned funerals, processions, sale of clothing, and gatherings in public assemblies, all of which reduced trade opportunities, and imposed severe penalties when those rules were not followed. Community bonds might be destroyed if people lose the opportunity to, for example, grieve, pay final respects, or assemble^1^. During the current pandemic, studying social mobility is essential. At the start of a pandemic and during the current phase, there is no real treatment or vaccination, which means that citizens need to rely on precautionary behaviour which is influenced by their risk attitudes. As the reality of the COVID-19 outbreak emerged, we saw that states started to introduce social distancing and isolation measures to deal with the pandemic and the lack of a vaccine. Even prior to that, individuals started adjusting their mobility behaviour.

## Hypotheses

We hypothesise that more risk-averse individuals are more likely to respond and less likely to engage in the behavioural changes which reduce risk. We also hypothesise that more risk-tolerant regions are less responsive to global announcements—in particular, their behaviour changes less to the COVID-19 outbreak classification as a pandemic by the WHO. We are also interested in comparing situations with higher or lower opportunity costs of human mobility. The opportunity costs of staying home (reduced mobility) are defined as the cost incurred by not enjoying the benefit of going out (benefits associated with the best alternative choice). For this, we explore differences between weekdays and the weekend. As many individuals are still working during the week, even while being at home, there is more psychological and potentially social pressure to be active during the weekend, which increases the opportunity costs of staying at home. Not going out requires to act against previously formed habits which is difficult for some. We therefore hypothesise that regions with higher risk tolerance are less likely to follow precautionary strategies when opportunity costs are higher and are therefore are less likely to deviate from their outside activities during the weekend relative to the baseline. Lastly, we also examine whether people adjust their behaviour when living in a population with a larger proportion of older people at greater risk of more severe illness or death from contracting the virus. We expect that regions with a higher share of over 65 individuals would show a greater reduction in mobility. In particular, risk-averse regions may display stronger mobility deviations from their original baseline (stronger reduction).

## Data and empirical strategy

We examined the relationship between the changes in human mobility during the outbreak of the novel Coronavirus disease (COVID-19) and the average risk preferences of individuals in 58 countries (with 776 regions from 33 countries with subnational regions data). Our main goal is to see if individuals in areas with higher (lower) levels of willingness to take risks are less (more) likely to reduce their exposure to social interactions by going to public places between 15 February 2020 and 9 May 2020. The outcome variables measure the daily *changes* (in percentage) in location visits compared to the median value of the same day of the week in the 5-week baseline period, between 3 January and 6 February 2020. To see whether mobility changes are related to risk tolerance, we first regressed each of the six mobility measures namely, *Retail & Recreation*, *Grocery & Pharmacy*, *Parks*, *Transit Stations*, *Workplaces*, and *Residential,* on risk-taking preference. In each regression, we controlled for whether the day is a weekend, an indicator distinguishing our sample period by the day when the World Health Organization (WHO) declared the COVID-19 outbreak a pandemic (11 March 2020), the total number of confirmed cases per 1000 people, number of days since the first confirmed coronavirus-related death in the country (days with no deaths (or before a death occurred) are coded 0), percentage of the population over 65, population density (per squared km of land area), percentage of urban population, average household size, unemployment rate, per capita income (in logs), daily average temperature, and a set of indicators on government responses that covers recommending and requesting closure of schools, workplace, public transport, stay at home, cancellation of public events, and restriction on gatherings and internal movement^43^. Consequently, our results regarding risk attitudes can be interpreted as independent of government lockdown measures. To this end, we employed a random-effects linear model to estimate the linear effect of risk-preference on mobility and linear interaction effects of risk and other covariates, namely, pandemic declaration, weekend, and the share of the population over 65.

## Results

**Figure 1:**
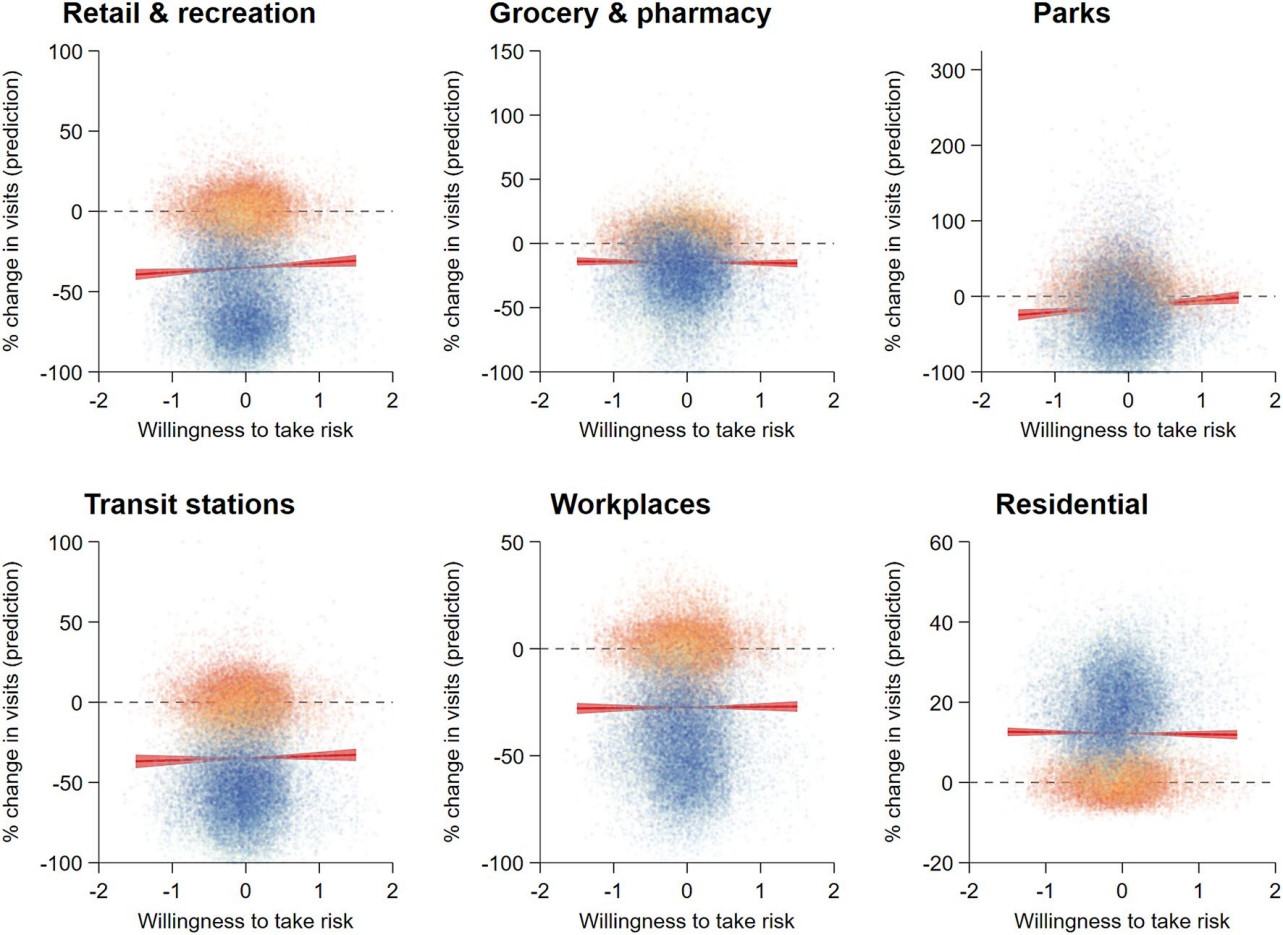
Risk attitude and human mobility during COVID-19. The six panels show the predicted percentage change in visit to locations classified as retail and recreation (*β* = 2.873, s.e. = 1.180, CI95% = [0.561;5.185], *P* = 0.015), grocery and pharmacy (*β* = − 0.481, s.e. = 1.060, CI_95%_ = [− 2.559;1.597], *P* = 0.650), parks (*β* = 7.667, s.e. = 2.577, CI_95%_ = [2.616;12.718], *P* = 0.003), transit stations (*β* = 1.352, s.e. = 1.350, CI_95%_ = [− 1.294;3.998], *P* = 0.317), workplaces (*β* = 0.306, s.e. = 0.848, CI_95%_ = [− 1.355;1.967], *P* = 0.718), and residential (*β* = − 0.241, s.e. = 0.374, CI_95%_ = [− 0.973;0.492], *P* = 0.519), compared to the respective baseline values over average individual risk preference. Estimates of the risk-mobility relation are obtained from random-effects linear regression (Table S1). Markers represent the daily change in visits to the six locations for each region during the entire sample period, with different colours showing observations over time (from most blue (first week of the sample period) to yellow (middle of the sample period) to most red (last week of the sample period)). For visualisation purpose, we excluded the Jammu and Kashmir (India) region.

We observe an overall reduction in visits (mobility) to all localities, other than residential places, for almost all regions, particularly in the earlier weeks in the sample period (see Fig. 1). A large proportion of observations showed an increase in visits to parks, even in the earlier phase, which might have been considered a relatively safe environment. Examining the general relationship between risk attitudes and the change in mobility in the entire sample period, we find some evident relationship to two locations. Particularly, risk-taking is positively associated with the change in visitation to places classified as retail and recreation (*β* = 2.873, s.e. = 1.180, CI_95%_ = [0.561;5.185], *P* = 0.015) and parks (*β* = 7.667, s.e. = 2.577, CI_95%_ = [2.616;12.718], *P* = 0.003), which indicates that in areas with higher average risk-tolerance, an individual is more likely to visit these places (or less likely to reduce their frequency of visits). On the other hand, there is no apparent relationship between risk preference and change in mobility to grocery and pharmacy (*β* = − 0.481, s.e. = 1.060, CI_95%_ = [− 2.559;1.597], *P* = 0.650), transit stations (*β* = 1.352, s.e. = 1.350, CI_95%_ = [− 1.294;3.998], *P* = 0.317), workplaces (*β* = 0.306, s.e. = 0.848, CI_95%_ = [− 1.355;1.967], *P* = 0.718) and residential areas (*β* = − 0.241, s.e. = 0.374, CI_95%_ = [− 0.973;0.492], *P* = 0.519).

Most control variables report the expected effect on change in human mobility. Specifically, there is a reduction in outings and an increase in staying home as severity increases, such as after the WHO declared the coronavirus outbreak a global pandemic, and with increases in the number of case per population (except for parks and residential, in which the relationship is positive and significant at 10% level and not significant, respectively), and most lockdown measures (see Supplementary Table S1). We also find that, on average, there is a greater reduction in visits to retail and recreational places (*β* = − 4.386, s.e. = 0.132, CI_95%_ = [− 4.645; − 4.127], *P* < 0.001), grocery and pharmacy (*β* = − 3.969, s.e. = 0.167, CI_95%_ = [− 4.295; − 3.642], *P* < 0.001), parks (*β* = − 4.543, s.e. = 0.446, CI_95%_ = [− 5.417; − 3.669], *P* < 0.001), and transit stations (*β* = − 0.791, s.e. = 0.189, CI_95%_ = [− 1.162; − 0.421], *P* < 0.001) on the weekends, in contrast to weekdays, while at the same time a reduction in visits to workplaces (*β* = 8.277, s.e. = 0.215, CI_95%_ = [7.855;8.698], *P* < 0.001) and staying home (*β* = − 3.284, s.e. = 0.110, CI_95%_ = [− 3.501; − 3.068], *P* < 0.001) is stronger on weekdays, compared to weekends. We note that while the number of days since the first death in the nation decreases significantly with going to transit stations (*β* = − 0.387, s.e. = 0.138, CI_95%_ = [− 0.658; − 0.117], *P* = 0.005), it had no effect or positive effect on mobility to other localities. Decline in visits to grocery and pharmacy (*β* = − 0.715, s.e. = 0.141, CI_95%_ = [− 0.992; − 0.438], *P* < 0.001), transit stations (*β* = − 0.304, s.e. = 0.166, CI_95%_ = [− 0.629;0.022], *P* = 0.068), and workplaces (*β* = − 0.361, s.e. = 0.092, CI_95%_ = [− 0.542; − 0.180], *P* < 0.001) is stronger for countries with a higher population density.

### Does the pandemic declaration increase the effect of risk attitudes?

We examine the interaction between willingness to take risks and the declaration of the pandemic. We find evidence suggesting the declaration of the pandemic is a strong moderator of the risk-mobility effect. It is relevant to note that the declaration of the pandemic precedes the lockdown measures of most governments.

**Figure 2:**
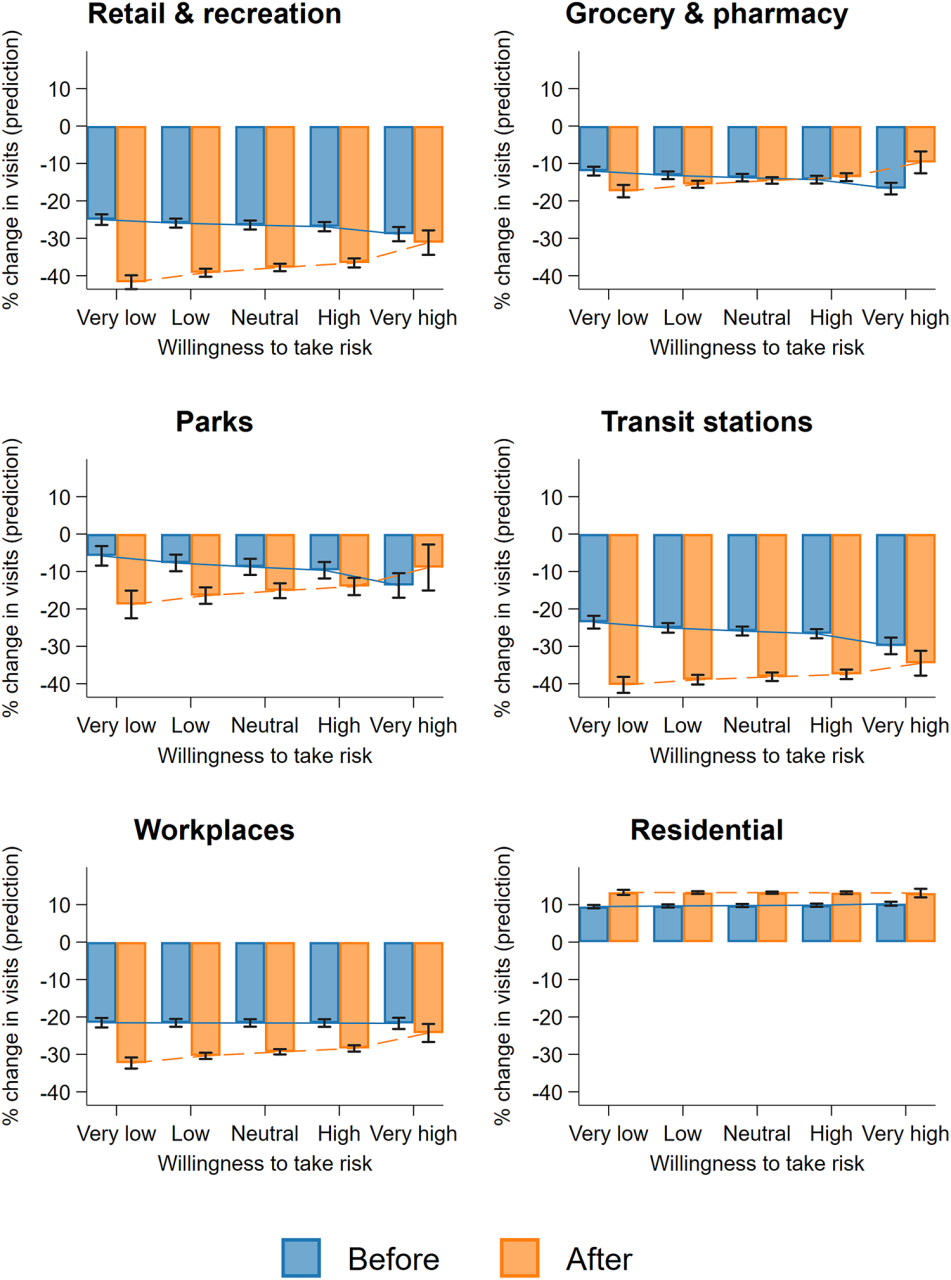
Change in visits to six location categories predicted by average individual risk preference before and after pandemic declaration. The six panels show the predicted percentage change in visit to locations classified as retail and recreation, grocery and pharmacy, parks, transit stations, workplaces, and residential, compared to the respective baseline values, before and after WHO declared COVID-19 as a pandemic on 11 March 2020, over average individual risk preference. Estimates are obtained from Table S2, for illustration, predicted changes are calculated over five points of the risk-taking variable (at the 1st, 25th, 50th, 75th, and 99th percentiles of the distribution), which we categorised into five levels of willingness to take risk: *very low*, *low*, *neutral*, *high*, and v*ery high*, respectively.

We observed that the reduction in outdoor activities (or increase in staying home) can be observed before COVID-19 was declared a pandemic by the WHO, especially for visits to places classified as retail and recreation, transit stations, and workplaces (see Fig. 2). The magnitude of mobility change has indeed increased after the declaration. For example, there is a further 11.3 percentage point (pp) reduction in visits to retail and recreation locations (*β* = − 11.328, s.e. = 0.879, CI_95%_ = [− 13.051; − 9.606], *P* < 0.001), 7.3 pp reduction in going to parks (*β* = − 7.303, s.e. = 1.379, CI_95%_ = [− 10.006; − 4.600], *P* < 0.001), 12 pp drop in going to transit stations (*β* = − 11.998, s.e. = 0.833, CI_95%_ = [− 13.631; − 10.365], *P* < 0.001), and 8 pp decrease in going to workplaces (*β* = − 8.103, s.e. = 0.642, CI_95%_ = [− 9.361; − 6.846], *P* < 0.001), respectively, compared to the period before pandemic declaration (Fig. 2, Table S1). In contrast, we find an average of 3.6 pp increase in staying in a residential area (*β* = 3.602, s.e. = 0.285, CI_95%_ = [3.042;4.161], *P* < 0.001) after declaration. Interestingly, the pandemic declaration did not have a severe impact on visits to grocery stores and pharmacies (*β* = − 0.987, s.e. = 0.705, CI_95%_ = [− 2.368;0.395], *P* = 0.162). Nonetheless, the findings in our robustness checks (Table S8) suggest that visits to grocery and pharmacy also decreased significantly after the pandemic declaration, which is in line with the estimate obtained from Table S1.

We find that, with respect to risk preferences, the changes to visitation patterns (compared to their respective baselines) are relatively greater for areas with lower average willingness to take risk, following the pandemic declaration. Specifically, we find the reduction in visits to grocery and pharmacy, transit stations, and workplaces *prior* to the declaration to be negatively correlated with the willingness to take risks. However, interrogating the interaction terms between risk-taking and pandemic declaration revealed a more interesting behavioural pattern; that is, the additional reduction in out-of-home activities after the declaration is much more dramatic for areas with more risk-tolerant individuals. We found a statistically significant interaction effect for each of the outcome variables except for residential places (retail and recreation: *β* = 6.715, s.e. = 1.166, CI_95%_ = [4.430;9.001], *P* < 0.001; grocery and pharmacy: *β* = 5.983, s.e. = 1.013, CI_95%_ = [3.998;7.968], *P* < 0.001; parks: *β* = 11.910, s.e. = 2.449, CI_95%_ = [7.110;16.711], *P* < 0.001; transit stations: *β* = 7.168, s.e. = 1.422, CI_95%_ = [4.381;9.954], *P* < 0.001; workplaces: *β* = 4.020, s.e. = 0.871, CI_95%_ = [2.313;5.726], *P* < 0.001; see Fig. 2). It is also important to note that pre- and post-declaration changes in visitation pattern differences are smaller for higher risk-tolerance areas and vice versa, indicating that areas with higher average risk-taking are less likely to respond to the negative change in environmental status.

### Mobility patterns on weekdays vs. weekends

**Figure 3:**
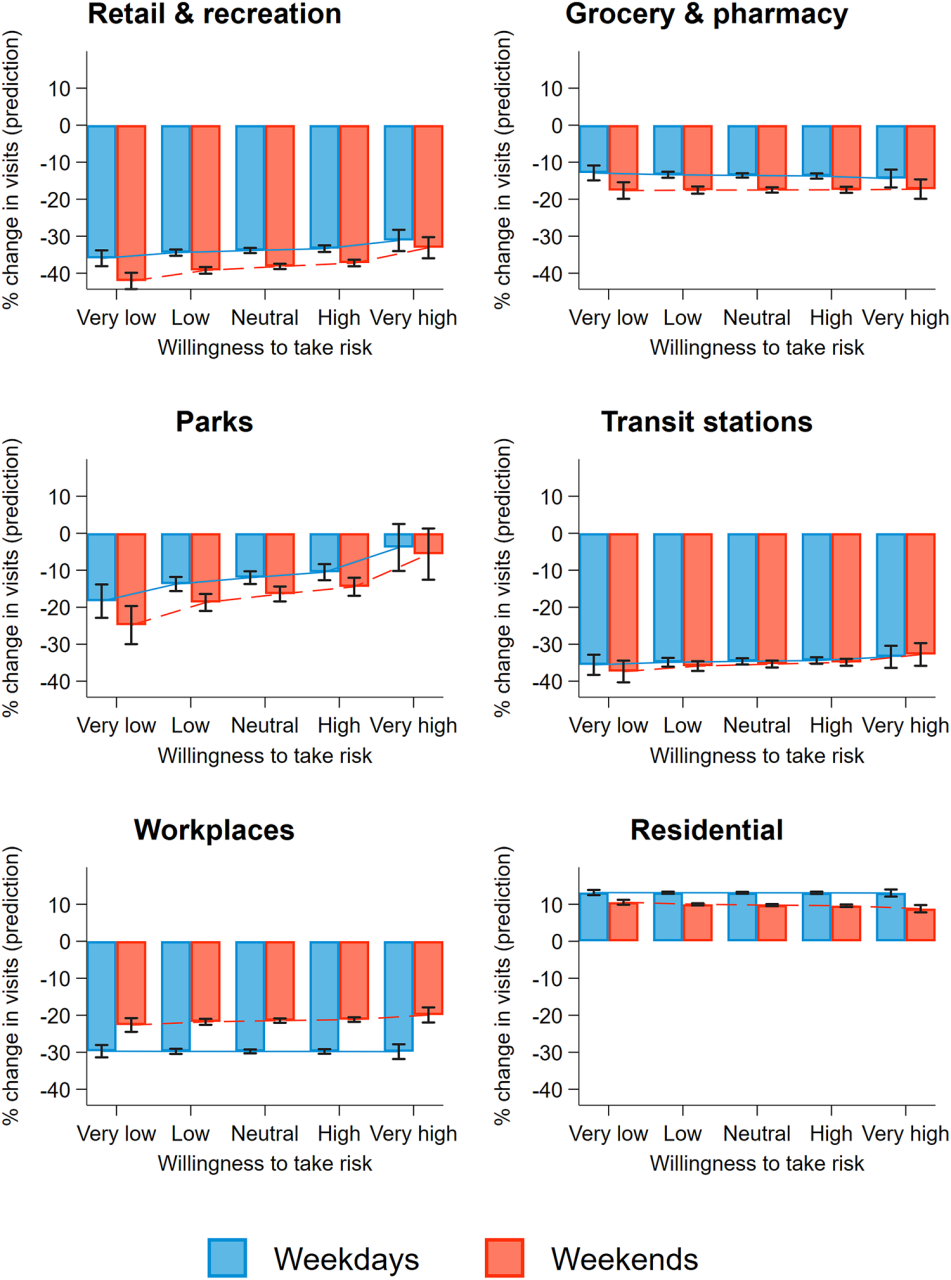
Visitation pattern by weekdays and weekends over average individual risk preference. The six panels show the predicted percentage change in visits to locations classified as retail and recreation, grocery and pharmacy, parks, transit stations, workplaces, and residential, compared to the respective baseline values in weekdays and weekends, over average individual risk-preference. Estimates are obtained from Table S3; for illustration, predicted changes are calculated over five points of the risk-taking variable (at the 1st, 25th, 50th, 75th, and 99th percentiles of the distribution), which we categorised into five levels of willingness to take risks: *very low*, *low*, *neutral*, *high*, and v*ery high*, respectively.

Next, we examine whether the tendency to change the frequency of visits to different localities during weekdays and weekends is mediated by risk attitudes. As Fig. 3 shows, our earlier results are confirmed. Compared to weekdays, individuals on average further reduce their visits to places (compared to the same day of the week in the baseline period) classified as retail and recreation by 4.3 pp (*β* = − 4.272, s.e. = 0.130, CI_95%_ = [− 4.527; − 4.017], *P* < 0.001; see Fig. 3), grocery and pharmacy by 3.9 pp (*β* = − 3.915, s.e. = 0.164, CI_95%_ = [− 4.236; − 3.594], *P* < 0.001), parks by 4.4 pp (*β* = − 4.392, s.e. = 0.450, CI_95%_ = [− 5.273; − 3.511], *P* < 0.001), and transit stations by 0.7 pp (*β* = − 0.723, s.e. = 0.185, CI_95%_ = [− 1.085; − 0.361], *P* < 0.001), compared to the baseline. In contrast, the reduction in going to workplaces is larger during weekdays (*β* = 8.342, s.e. = 0.213, CI_95%_ = [7.925;8.759], *P* < 0.001). While individuals are more likely to stay home (places classified as residential) in general, the (percentage point) increase of staying home is higher during weekdays compared to weekends (*β* = − 3.346, s.e. = 0.109, CI_95%_ = [− 3.560; − 3.131], *P* < 0.001). The coefficients of the interaction terms provide strong evidence that regions with lower risk tolerance have a larger reduction in mobility during weekends than in weekdays, compared to those who are more risk-tolerant (retail and recreation: *β* = 2.011, s.e. = 0.318, CI_95%_ = [1.388;2.634], *P* < 0.001; grocery and pharmacy: *β* = 0.916, s.e. = 0.411, CI_95%_ = [0.110;1.722], *P* = 0.026; parks: *β* = 2.261, s.e. = 1.015, CI_95%_ = [0.272;4.250], *P* = 0.026; transit stations: *β* = 1.181, s.e. = 0.502, CI_95%_ = [0.197;2.165], *P* = 0.019; workplaces: *β* = 1.375, s.e. = 0.506, CI_95%_ = [0.384;2.367], *P* = 0.007; residential: *β* = − 0.789, s.e. = 0.260, CI_95%_ = [− 1.298; − 0.279], *P* = 0.002). Results from robustness checks also confirm our findings (see Table S9 in SI Appendix).

**Figure 4:**
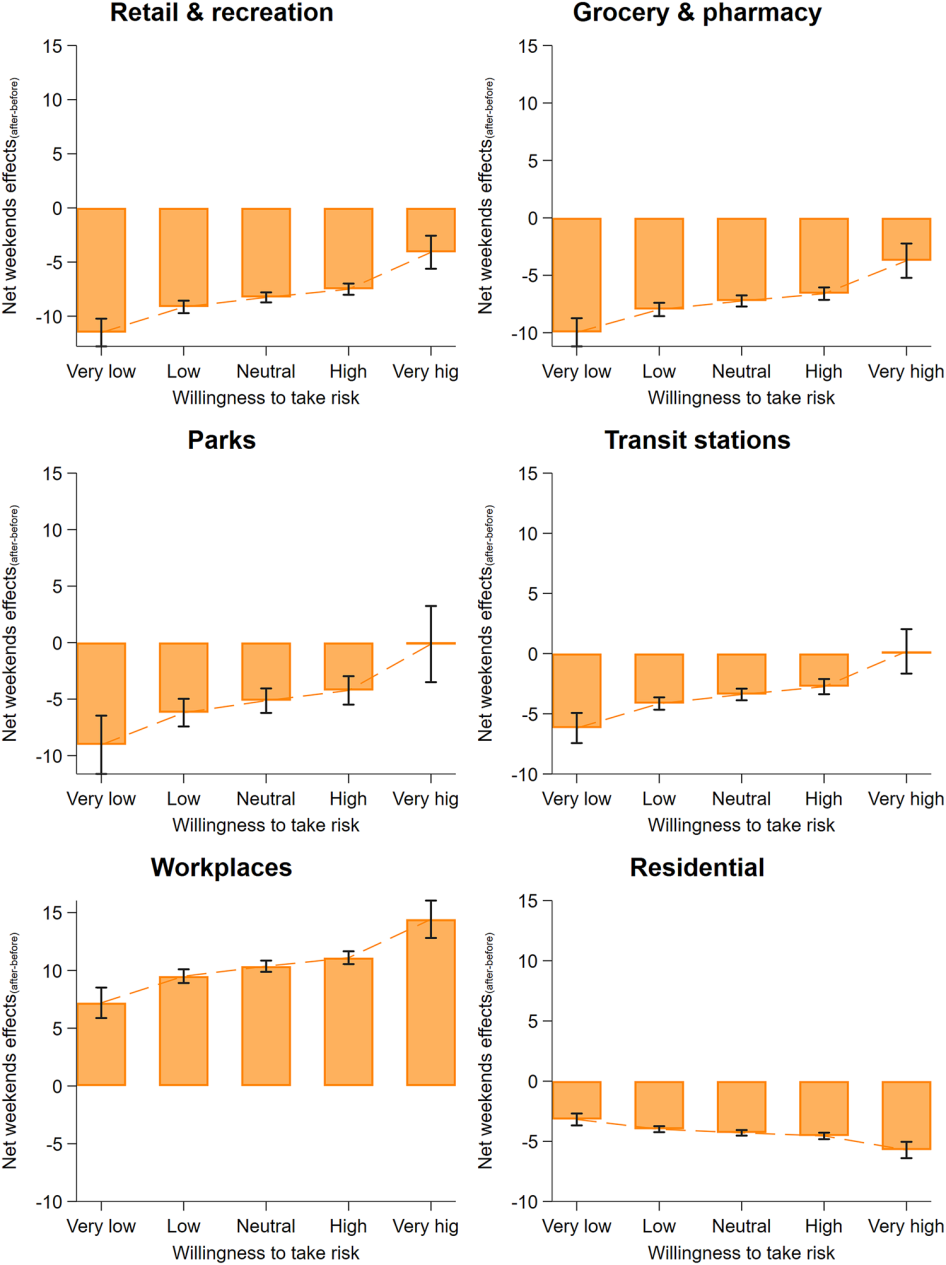
Mediation from risk preference to change in weekends and weekdays visiting pattern is stronger after pandemic declaration. The six panels show the difference in average marginal effects of weekends on visits to locations classified as retail and recreation, grocery and pharmacy, parks, transit stations, workplaces, and residential pre- and post-pandemic declaration periods, over risk-tolerance levels. Estimates are obtained from Table S4; for illustration, predicted changes are calculated over five points of the risk-taking variable (at the 1st, 25th, 50th, 75th, and 99th percentiles of the distribution), which we categorised into five levels of willingness to take risks: *very low*, *low*, *neutral*, *high*, and v*ery high*, respectively.

Moreover, we find that the mediation effect is more apparent after the pandemic declaration, suggesting the effect manifests alongside severity. Specifically, we reran the analysis by including the interaction between the risk preference-weekend mediation effect and pandemic declaration dummy (triple interaction term). We visualised the results in Fig. 4, showing the difference in average marginal effects of weekends (in contrast to weekdays) before and after the pandemic announcement, over levels of risk-taking (pre- and post-declaration average marginal effects of weekends is shown in Fig. S1 and predicted change in mobility in Fig. S2). We find that the tendency to reduce going out during the weekends compared to weekdays increases significantly with the levels of risk-tolerance for all non-residential and work locations, particularly in the post-declaration period (retail recreation: *β* = 5.036, s.e. = 0.707, CI_95%_ = [3.651;6.421], *P* < 0.001; grocery pharmacy: *β* = 4.273, s.e. = 0.698, CI_95%_ = [2.904;5.642], *P* < 0.001; parks: *β* = 5.989, s.e. = 1.532, CI_95%_ = [2.985;8.993], *P* < 0.001; transit stations: *β* = 4.697, s.e. = 0.884, CI_95%_ = [2.965;6.429], *P* < 0.001). It can also be seen that regions with higher risk-taking attitude have a larger pre-post-declaration relative weekends-weekdays difference in mobility for workplaces (*β* = 4.008, s.e. = 0.665, CI_95%_ = [2.705;5.312], *P* < 0.001) and residential places (*β* = − 1.397, s.e. = 0.290, CI_95%_ = [− 1.966; − 0.829], *P* < 0.001). These results are highly robust to our checks (see Fig. S3 and Table S10 in SI Appendix).

### Actual risk

**Figure 5:**
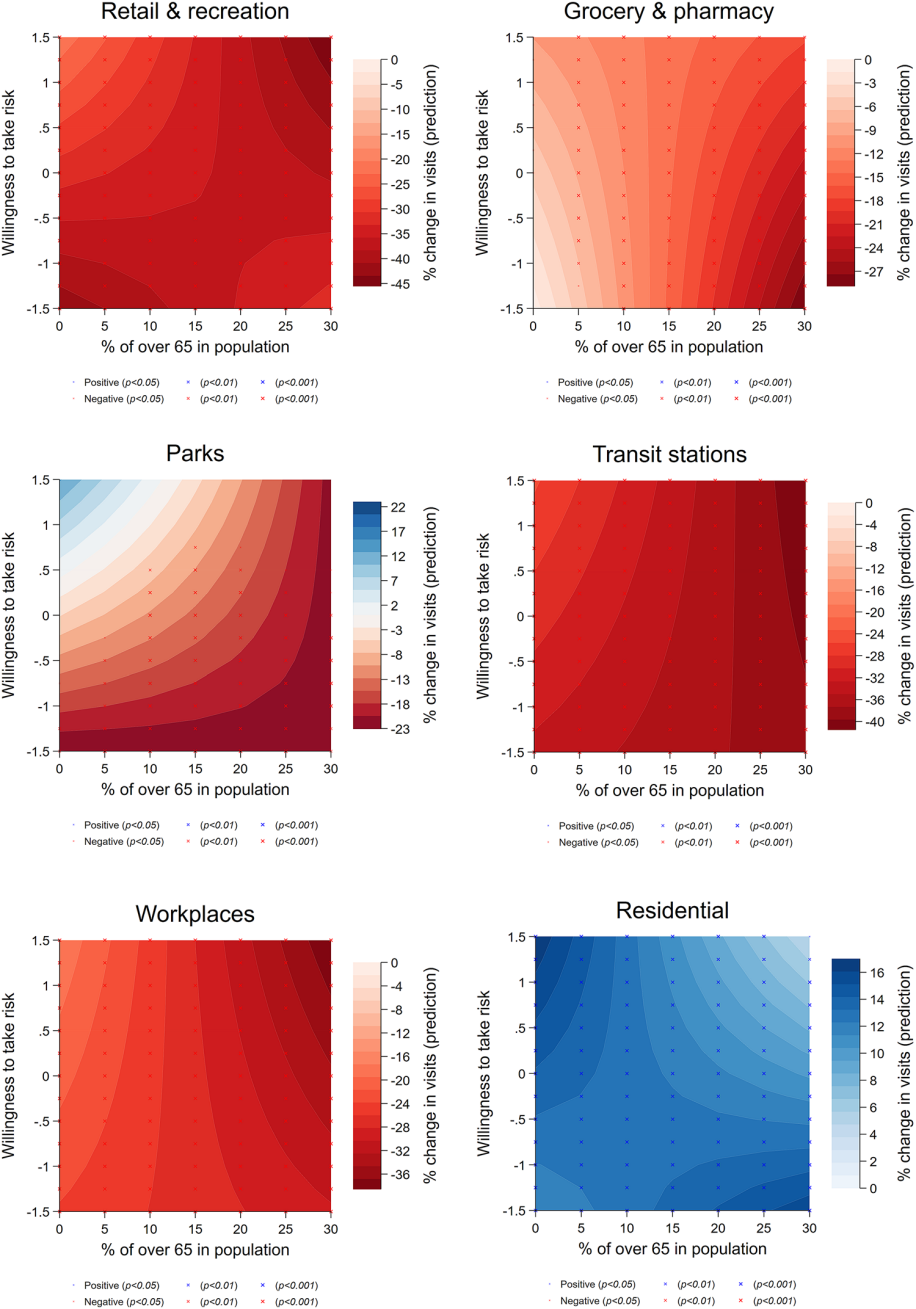
Change of mobility patterns based on risk preference and share of the population over 65. The six panels show the predicted change in visits to locations classified as retail and recreation, grocery and pharmacy, parks, transit stations, workplaces, and residential, over risk-tolerance levels and the proportion of 65 + in the population. Estimates are obtained from Table S5.

Next, we examine the relationship between mobility changes, risk attitudes, and proportion of elderly in the population to test if the relationship between mobility and risk is moderated by the share of the population at higher risk of dying from COVID-19. We thus regressed change in mobility on willingness to take risk and share of the population over 65 and the interaction between the two (see Fig. 5). We found that areas with a larger population at fatal risk (elderly) have larger cutback in going to grocery and pharmacy (*β* = − 0.597, s.e. = 0.164, CI_95%_ = [− 0.918; − 0.277], *P* < 0.001), transit stations (*β* = − 0.364, s.e. = 0.153, CI_95%_ = [− 0.664; − 0.063], *P* = 0.018), and workplaces (*β* = − 0.447, s.e. = 0.096, CI_95%_ = [− 0.636; − 0.258], *P* < 0.001), as well as a decrease in staying at home (*β* = − 0.128, s.e. = 0.049, CI_95%_ = [− 0.224; − 0.033], *P* = 0.009)*,* even though the size of the coefficients suggest the magnitude of the effect is quite small (e.g., with 1 pp increase in share of over 65′s in population, mobility change for staying home decreases by 0.1 pp). While we did not find a significant (negative) change in mobility to retail and recreation (*β* = − 0.226, s.e. = 0.148, CI_95%_ = [− 0.517;0.065], *P* = 0.128) and parks (*β* = − 0.561, s.e. = 0.484, CI_95%_ = [− 1.511;0.388], *P* = 0.247) due to population risk level, results from the robustness checks show the negative effect is significant (see Table S11). Moreover, we found a significant interaction effect on mobility of retail and recreation (*β* = − 0.388, s.e. = 0.169, CI_95%_ = [− 0.719; − 0.056], *P* = 0.022) and residential (*β* = − 0.183, s.e. = 0.050, CI_95%_ = [− 0.281; − 0.086], *P* < 0.001). This suggests that in areas with more risk-loving individuals and a larger proportion of the population at risk, people seem to have further reduced going to retail and recreation places; however, regions with fewer risk-takers and larger older population increase their time staying home. Nonetheless, for mobility changes in other localities, the effect of risk-taking attitudes does not seem to be moderated by the actual population risk factor.

## Discussion

### Summary

There are stark differences between how we perceive risk and the reality or the calculated level of risk, which can result in very different behavioural responses. Risk attitudes are shown to shape the observable behavioural responses to pandemics. The actual (calculated) health risks of the COVID-19 pandemic are comparatively low for most age groups apart from the elderly^44–46^. Nevertheless, risk attitudes—rather than actual risk—influence the real behavioural activity for the young and the elderly. This is particularly important as changes in risk perceptions may be induced by more information about the disease. If relevant parts of the population do not perceive the risk of COVID-19 to themselves as very threatening, their behavioural responses are likely to induce further spread of the virus.

### Pandemic declaration

Our results demonstrate the sharp shifts in the relation between behavioural activity and risk attitudes before and after the declaration of COVID-19 as a pandemic, as well as shifts before and after the first related death was recorded in each region. The first thing that becomes apparent is that behaviour and our willingness to take on risks have both shifted dramatically since the baseline period in mid-February. At this time, only three deaths were recorded outside mainland China (one in each of Hong Kong, Japan, and the Philippines) and life was largely proceeding as normal. There was no imminent perception of a threat or of the worldwide pandemic to come, reflected in the baseline reporting of behaviour and the willingness to take risks. However, when we compare this to the first and second sample period, we observe mostly negative shifts in behaviour (excluding residential) but a mixed set of reactions to risk. Several categories saw a substantial negative shift in visits, including Retail & Recreation, Transit Stations, and Workplaces; compared to the baseline, visiting behaviours had already started to drop off before the pandemic announcement and dropped off more sharply afterwards. During this first period, we can see that social distancing and work from home was starting to make an impact, as people stopped travelling to and from work (especially through crowded transit stations) and also stopped engaging in non-essential retail shopping. After the pandemic was officially announced, we see a second wave of behavioural shifts as more people reduce their travel, shopping and more either lose their jobs or are in lockdown. However, we do observe an interesting shift in how risk attitudes affect mobility across these three categories as they all exhibit a slightly positive trend in the period before the announcement, but they all shift to a much stronger negative risk trend after the announcement. Given that ‘flattening the curve’ was the strategic focus for most governments, the social distancing message appears to have been received even prior to most lockdown measures. Conversely, Grocery & Pharmacy, Parks, and Residential had much smaller shifts both before and after the announcements when compared to the baseline. However, the shifts in Grocery & Pharmacy and Parks—while much smaller than the other categories—appear to undergo a large risk preference-mobility shift; that is, the first set of behavioural changes results in a positive sloping risk function that flipped into a negative sloped function after the pandemic announcement.

While seemingly at odds with expectations, one may want to consider what the announcement of the pandemic would have meant to most individuals. With a looming threat of lockdown and isolation, at this point, individuals would have ramped up shopping to stock up for likely upcoming government lockdowns. In addition, those with an affinity for the outdoors may have wanted to enjoy their parks and outdoor lifestyle as much as possible before it was banned. This is in line with the reported shifts in the number of visits, which while still negative overall, indicate that the change to number of visits is less negative than before the announcement.

### Weekdays vs. weekends

When interpreting these statistics, we need to bear in mind the ‘normal’ weekly habits of people; that is, working during the week and undertaking other activities/pastimes on the weekends. In order to ensure we capture the shift in behaviour, we compare the weekday behaviours and risk attitudes to that of the weekends. Our result demonstrates that there are differences between weekdays and weekends, as one would expect that on weekends there are slightly more activities taking place other than work. Furthermore, we see little variance in the slopes of weekdays and weekends risk attitudes. The large negative shifts across all categories except workplaces after the official declaration, but much smaller variations between weekdays and weekends before the declaration, further supports the discussion above: that the behaviour had already started to change well before the declaration of a pandemic, with many individuals starting to increase their weekend activities. However, after the pandemic was announced, a raft of measures that tried to limit the spread of the virus resulted in a very large change in most economies due to the closure of businesses and job losses. This fundamental change in economic activities and loss of work left very little to differentiate weekends from weekdays for a large number of people, which is reflected in the large negative changes in the comparisons. Prior to the announcement, we see that the function on willingness to take risk is fairly flat or slightly downward sloping, but risk perceptions change significantly for all categories after the announcement. The most interesting changes occurred in Workplace and Residential mobility, exhibiting a relatively large increase in the willingness to take high risks: this could be explained through people wanting to visit family and friends or the increased willingness to work despite the risk of infection.

### Behavioural stability

In general, throughout our analysis, we observe that less risk-tolerant regions more actively adjust their behavioural patterns in response to the pandemic. Risk-seeking regions are less responsive to protective measures. Thus, the tendency towards being more or less cautious carries substantial behavioural implications that are also affected by different levels of opportunity costs, as evidenced by the weekend effect. Regional differences seem to matter, offering support for a “regional personality factor” in risk-taking.

Risk-takers, therefore, seem to demonstrate a lower preference for their own and communal safety, as demonstrated by the fact that risk-averse regions with higher percentages of 65 + people are more actively increasing social isolation by staying at home. Such behavioural differences due to risk preferences may indicate different levels of homeostatic responses. Risk aversion seems to promote a stronger fluctuation around a target level. For example, if you are driving on a motorway and it starts to rain or snow, what do you do? Our result would imply that risk-averse individuals may be more likely to slow down to reduce the likelihood of having an accident. Risk-averse individuals have a higher need for risk compensation. Thus, the level of risk at which a person feels best is maintained homeostatically in relation to factors such as emotional or physiological experiences^26^.

Overall, the lack of adjustment among risk-taking regions is interesting, as many settings that explore risk-taking behaviour are connected to the possibility of attracting social fame and praise, financial gains, or other potential positive outcomes. In our setting, risks are strongly attached to the loss health or life (their own and other’s) without achieving major gains, although positive utility gains also arise from not restricting one’s usual activities. It seems like the risk-takers are more behaviourally stable during such environmentally challenging circumstances. It is almost as if risk-taking regions are more determined to maintain settings as activity-oriented, while risk-averse regions are more goal-oriented in achieving social distancing.

### Cognitive re-evaluation

The current analysis is interesting, as a large number of studies exploring the implications of risk are based on cross-sectional samples or between-subject designs in laboratory settings. In this case, the danger is more prolonged, lasting over several weeks or months, compared with other risk situations such as driving a car. Automatic or response “scripts” become less relevant as individuals have the chance to think about their actions and adjust their behaviour accordingly. Strategic, tactical, or operational factors become more dominant, while perceptual, emotional, and motivational factors remain active. In addition, individuals do not face a single “either-or” decision but are required to constantly evaluate their choices to go out or stay at home. Thus, cognitive re-evaluation is a core feature in our setting and is based on dynamic feedback loops. However, humans are very social animals, and over time, the lack of social interaction is likely to erode some individual’s willingness to comply with social distancing or lockdown regulations. This is likely to trigger a re-evaluation of how people perceive the risk of transmission and the perceived benefits of maintaining their lower levels of mobility. Risk-loving regions are also less likely to adjust their behaviour based on external stimuli, such as the WHO announcement of classifying COVID-19 as a pandemic.

### Limitations and avenues for future research

A core limitation of this study is that we are only able to explore human behaviour at the regional and not individual level. Studies that use individual data could focus in more detail on individual differences such as age, gender, or differences in affective reactions or perceived locus of control and could try to disentangle perceptions (risk preferences) partly from actual risk as statistics provide detailed information on the actual age-adjusted risk profile. Such a study would provide a better understanding of habit changes, as well as potentially reveal motivational reasons for behavioural changes or behavioural stickiness. To reduce levels of uncertainty or ambiguity, individuals will try to gain control over a situation or they will change their preferences to better the fit the situation, and thus try to gain control in a secondary way^26^. Other psychological factors, such as overconfidence may also matter. In addition, we do not have information about the actual level of social mobility in the baseline period. If that information were available, one could argue that those who had the highest levels of mobility prior to the lockdown have had the largest relative loss; we should therefore observe this group exhibiting the most risk-seeking behaviour and breaking the lockdown rules. On the other hand, those who previously had the least amount of social mobility have in relative terms only suffered a small loss—and should be much less likely to break the lockdown rules. However, this may adjust over time, as individuals habituate to the changes and reset their reference points. This fits nicely into the suggestion that “a person who has not made peace with his losses is likely to accept gambles that would be unacceptable to him otherwise” [^37^, p. 287], which is consistent with risk preference changes in a disaster situation^13^.

Risk is a fascinating topic as we have two forces in place. Based on evolutionary theory, people are risk-inclined but also control-inclined. Risk-taking is necessary to cope with environmental changes and the constant level of uncertainty and danger. On the other hand, control of the environment is required to reduce risks that go beyond the desired levels or that may pose a danger to one’s survival^26^.

The pandemic declaration caused a fundamental shift in human behaviour around the world, independent of any government lockdown measures. Future studies should explore in more detail how information dissemination and media reporting are connected to behavioural responses and the level of risk-taking within regions. Removal of the lockdown policies is likely to be undertaken cautiously and slowly rather than via one large change. It is unclear at this stage how changes—particularly among the risk-averse regions—have already led to new habit formation that will not readjust to previously normal settings. Future studies will provide more insights into such a question.

## Materials and methods

### Mobility

We obtained the mobility measures at the country and regional level from the COVID-19 Community Mobility Reports^47,48^, accessed on 16 May 2020. The dataset consists of six location-specific mobility measures for 132 countries between 15 February 2020 and 9 May 2020. For 51 out of the 131 countries, the mobility measures are also available at the regional level (For Finland, Great Britain, Slovenia, and Latvia, regions are identified as the second-level administrative divisions while the rest are first-level administrative divisions). For the United States, both state and county-level data are available, although we did not include county level in our analysis as risk preferences are not available at the county level. The resulting number of sub-national regions included is 1207. Based on anonymised and aggregated data from Google users who have opted into the Location History service, each mobility measure records the percentage change in visits and length of stay to places classified as *Retail & Recreation*, *Grocery & Pharmacy*, *Parks*, *Transit Stations*, *Workplaces*, and *Residential* within the geographic area. The per cent change is compared to the median value of the same day of the week between 3 January and 6 February 2020. For privacy reasons, Google censors values if traffic volumes are not high enough to ensure anonymity. While the median number of censored values for each mobility measure is zero, about 48% (*n* = 619) of regions have at least one censored value for any of the six mobility variables on any given day in the sample period. To ensure our results were not caused by the unbalanced sample due to censored values, we reran our results by excluding regions with various thresholds of daily values censored, finding that the results remain highly robust to all exclusions (see Table S7 to S11 in SI Appendix).

### Risk attitudes

We obtain the measure of risk preferences from the globally representative Global Preferences Survey collected in 2012 using the Gallup World Poll^16,17^, which is aggregated into the country (*n* = 76) and regional (*n* = 1126) level. Risk preferences of the respondents were elicited through a qualitative question (self-rated perceived risk preferences on an 11-point scale) and a set of quantitative questions using the staircase method, where respondents were asked to choose between varying sure payments and a fixed lottery, in which the individual could win amount X with some probability *p* or zero with probability 1—*p*. The responses from the two questions were combined (with roughly equal weights) to produce the overall individual risk preference measure^17^. For subnational regions where both mobility measures and risk preference measures are available at the regional levels, we employed the regional aggregated values (average of standardised values at the individual level); otherwise country aggregated values were used.

### COVID-19 cases and deaths statistics and government response indicators

Country-level statistics on the daily number of cases and deaths were sourced from the European Centre for Disease Prevention and Control (ECDC). Together with the set of indicators on government responses, these data were obtained from the Oxford COVID-19 Government Response Tracker (OxCGRT) (^43^, accessed on 18 May 2020), available for 167 countries recorded daily from 1 January 2020. Out of the 17 response indicators available from the OxCGRT, we take seven indicators on policies regarding social isolation and confinement, including school, workplace, and public transport closures, public events cancellations, stay at home requirements, and gatherings and internal movement restrictions. Each indicator has various levels, from no measures taken to recommendation and implementation of the policy, recorded on an ordinal scale. For example, workplace closure is classified into four levels (1—no measures; 2—recommend work from home; 3—require closing for some categories or sectors of workers; and 4—require closing for all-but-essential workplaces). We dichotomously coded each response to be included in our regression analysis. OxCGRT also records if the policy is applied nationwide; for robustness checks, we recode each response indicators as no measures taken if the policy is targeted to a specific geographical region (see SI Appendix).

### Control variables

#### Demographic indicators

Population density (people per squared km of land area), percentage of urban population, share of population ages 65 were obtained from the World Development Indicators^49^ and are available at the country level. We also collected the average household size (average number of usual residents per household) from the Database on Household Size and Composition 2019^50^. *Economic indicators.* We also obtained the latest available estimates (as of May 2020) of the unemployment rate (% of total labour force), based on estimates from the International Labour Organization, and per capita GDP (2010 US$ constant, in natural log form) from^49^. *Weekend*. We employ the definition of working week across countries according to^51^. *Daily average temperature*. Temperature data is obtained from the GHCN (Global Historical Climatology Network)-Daily database^52,53^, accessed on 19 May 2020. For each region, the daily average temperature (in tenths of degree Celsius (°C)) was calculated from taking the mean of the average temperature recorded from all weather stations located within 50 km from the centroid of the region. Using median temperature does not change our results.

### Combining datasets

To join datasets together for our analysis, we use regions defined in the Google Mobility dataset as our point of reference. In general, for regions with mobility measures but not from another dataset (i.e., risk attitudes or average daily temperature is unavailable for that region), we employ its country values. The resulting number of countries in our final sample is 58, after merging all variables used in this study, with a total of 776 subnational regions from 33 countries (see Table S6 in SI Appendix). The total number of region-day observations ranges from 58,284 to 67,073, depending on the availability of mobility measures.

### Analyses

To examine the main question of how mobility patterns during the COVID-19 outbreak change according to risk attitudes, we analysed the data using random-effects linear model. Standard errors are clustered on the smallest geographic unit in each regression. Data and codes used in this study can be found on the Open Science Framework (https://osf.io/7bxqp/).

## Supplementary information

Supplementary Information
