# Supplementary material for: Risk attitudes and human mobility during the COVID-19 pandemic
Source: Sci Rep. 2020 Nov 16;10:19931. doi: 10.1038/s41598-020-76763-2 (PMC7669857; doi:10.1038/s41598-020-76763-2)
Supplement: Supplementary file 1 — Supplementary Information [file 41598_2020_76763_MOESM1_ESM.pdf]

Supplementary Information for:

Risk Attitudes and Human Mobility during the COVID-19 Pandemic

Ho Fai Chan[1,2*], Ahmed Skali[3], David A. Savage[2,4], David Stadelmann[2,5,6,7], Benno Torgler[1,2,7]

[1]School of Economics and Finance, Queensland University of Technology, Australia
[2]Centre for Behavioral Economics, Society and Technology (BEST), Australia
[3]Department of Economics, Deakin University, Australia
[4]Newcastle Business School, University of Newcastle, Australia
[5]University of Bayreuth, Germany
[6]IREF – Institute for Research in Economic and Fiscal Issues
[7]CREMA—Center for Research in Economics, Management, and the Arts, Switzerland
*e-mail: hofai.chan@qut.edu.au

Supplementary Results

Robustness Checks.

This section presents the checks for robustness of our results, which are shown in Table S7 to S11 for the six sets of regressions conducted in the main text, respectively. The first two checks concern including regions with censored mobility value in the sample of the analysis. We impose two restrictions on sample inclusion 1) regions with at least one censored value for the outcome mobility measures are excluded from the corresponding regression and 2) a more restrictive criteria with regions at least one censored value for any of the outcome mobility measures are excluded from the analysis. The first criteria excluded number of regions ranging from 54 (Workplace) to 217 (Residential), depending on the outcome mobility measure used while the second criteria reduce the number of regions to 484. The third check concerns if estimates are sensitive to whether government response is general by recoding indicators as no measures taken if the movement restrictions (or recommendation of restrictions) were not applied countrywide. In general, our main findings are robust to all three checks.

For the overall risk-mobility relationship (comparing estimates from Table S7 to Table S1), imposing sample exclusions increases the strength of the relationship for all mobility measures except for transit station under the first exclusion rule (first exclusion rule: retail & recreation: β=2.219, s.e.=1.183, $CI_{95\%}$=[-0.099;4.537], P=0.061; grocery & pharmacy: β=-0.325, s.e.=1.006, $CI_{95\%}$=[-2.298;1.647], P=0.746; parks: β=8.019, s.e.=2.535, $CI_{95\%}$=[3.050;12.987], P=0.002; transit stations: β=1.306, s.e.=1.352, $CI_{95\%}$=[-1.344;3.956], P=0.334; workplaces: β=0.761, s.e.=0.854, $CI_{95\%}$=[-0.913;2.434], P=0.373; residential: β=0.089, s.e.=0.413, $CI_{95\%}$=[-0.721;0.900], P=0.829; second exclusion rule: retail & recreation: β=2.551, s.e.=1.074, $CI_{95\%}$=[0.447;4.656], P=0.017; grocery & pharmacy: β=-0.279, s.e.=0.905, $CI_{95\%}$=[-2.052;1.494], P=0.758; parks: β=9.463, s.e.=2.589, $CI_{95\%}$=[4.388;14.539], P<0.001; transit stations: β=1.865, s.e.=1.186, $CI_{95\%}$=[-0.459;4.189], P=0.116; workplaces: β=-0.246, s.e.=0.773, $CI_{95\%}$=[-1.761;1.269], P=0.751; residential: β=0.251, s.e.=0.427, $CI_{95\%}$=[-0.587;1.088], P=0.557). Transforming the government response indicators slight reduce the size of the coefficients while leaving the statistical significance unchanged (retail & recreation: β=3.724, s.e.=1.225, $CI_{95\%}$=[1.324;6.124], P=0.002; grocery & pharmacy: β=-0.247, s.e.=1.138, $CI_{95\%}$=[-2.477;1.984], P=0.828; parks: β=6.070,

s.e.=2.455, $CI_{95\%}$=[1.258;10.882], *P*=0.013; transit stations: *β*=1.597, s.e.=1.597, $CI_{95\%}$=[-1.534;4.727], *P*=0.317; workplaces: *β*=0.581, s.e.=0.940, $CI_{95\%}$=[-1.261;2.424], *P*=0.536; residential: *β*=-0.481, s.e.=0.374, $CI_{95\%}$=[-1.213;0.252], *P*=0.199). The coefficient estimates for our main control variables (*pandemic declaration* and *weekends*) are also close to those found in the main results, apart from *% population 65+*, where the negative effects on mobility change to non-residential places are more prominent in the restricted samples (checks 1 and 2).

For the declaration moderator effect on the risk-mobility relationship, the results (coefficients of the declaration x risk preference term) remain highly robust except for residential, where statistically significance is drop when regions with censored values were removed (first exclusion rule: Retail & recreation: *β*=4.965, s.e.=1.210, $CI_{95\%}$=[2.593;7.337], *P*<0.001; Grocery & pharmacy: *β*=6.265, s.e.=1.075, $CI_{95\%}$=[4.157;8.372], *P*<0.001; *P*arks: *β*=10.471, s.e.=2.477, $CI_{95\%}$=[5.617;15.325], *P*<0.001; Transit stations: *β*=6.919, s.e.=1.514, $CI_{95\%}$=[3.952;9.887], *P*<0.001; Workplaces: *β*=3.768, s.e.=0.880, $CI_{95\%}$=[2.042;5.494], *P*<0.001; Residential: *β*=-0.216, s.e.=0.515, $CI_{95\%}$=[-1.225;0.793], *P*=0.675; second exclusion rule: Retail & recreation: *β*=4.702, s.e.=1.388, $CI_{95\%}$=[1.981;7.422], *P*<0.001; Grocery *&* pharmacy: *β*=4.146, s.e.=1.190, $CI_{95\%}$=[1.815;6.478], *P*<0.001; *P*arks: *β*=11.653, s.e.=2.695, $CI_{95\%}$=[6.372;16.935], *P*<0.001; Transit stations: *β*=4.885, s.e.=1.698, $CI_{95\%}$=[1.556;8.213], *P*=0.004; Workplaces: *β*=1.504, s.e.=0.995, $CI_{95\%}$=[-0.446;3.453], *P*=0.131; Residential: *β*=-0.092, s.e.=0.548, $CI_{95\%}$=[-1.166;0.983], *P*=0.867; government response indicators transformed: Retail & recreation: *β*=2.983, s.e.=1.083, $CI_{95\%}$=[0.860;5.107], *P*=0.006; Grocery & pharmacy: *β*=2.926, s.e.=1.025, $CI_{95\%}$=[0.917;4.936], *P*=0.004; *P*arks: *β*=7.071, s.e.=2.382, $CI_{95\%}$=[2.403;11.740], *P*=0.003; Transit stations: *β*=2.934, s.e.=1.322, $CI_{95\%}$=[0.343;5.526], *P*=0.026; Workplaces: *β*=0.284, s.e.=0.923, $CI_{95\%}$=[-1.524;2.092], *P*=0.758; Residential: *β*=1.065, s.e.=0.443, $CI_{95\%}$=[0.197;1.934], *P*=0.016).

For the weekend x risk-taking interaction term, the coefficients for retail and recreation remain statistically significant in all three checks (first exclusion rule: *β*=1.622, s.e.=0.308, $CI_{95\%}$=[1.018;2.225], *P*<0.001; second exclusion rule: *β*=2.038, s.e.=0.349, $CI_{95\%}$=[1.354;2.722], *P*<0.001; government response indicators transformed: *β*=1.464, s.e.=0.331, $CI_{95\%}$=[0.814;2.113], *P*<0.001) as well as for parks (first exclusion rule: *β*=3.441, s.e.=1.092, $CI_{95\%}$=[1.301;5.581], *P*=0.002; second exclusion rule: *β*=4.474, s.e.=1.306, $CI_{95\%}$=[1.914;7.035], *P*<0.001; government response indicators transformed: *β*=1.997,

s.e.=1.026, $CI_{95\%}$=[-0.015;4.009], P=0.052), transit stations (first exclusion rule: β=1.251, s.e.=0.541, $CI_{95\%}$=[0.192;2.311], P=0.021; second exclusion rule: β=1.964, s.e.=0.585, $CI_{95\%}$=[0.817;3.112], P<0.001; government response indicators transformed: β=0.914, s.e.=0.494, $CI_{95\%}$=[-0.053;1.882], P=0.064), workplaces (first exclusion rule: β=1.316, s.e.=0.511, $CI_{95\%}$=[0.314;2.317], P=0.010; second exclusion rule: β=2.065, s.e.=0.635, $CI_{95\%}$=[0.820;3.310], P=0.001; government response indicators transformed: β=1.115, s.e.=0.494, $CI_{95\%}$=[0.147;2.083], P=0.024), and residential area (first exclusion rule: β=-1.141, s.e.=0.305, $CI_{95\%}$=[-1.739;-0.543], P<0.001; second exclusion rule: β=-1.255, s.e.=0.321, $CI_{95\%}$=[-1.884;-0.626], P<0.001; government response indicators transformed: β=-0.724, s.e.=0.259, $CI_{95\%}$=[-1.232;-0.215], P=0.005). Transforming government response indicators rendered the significance of the interaction effects for going to grocery and pharmacy (β=0.466, s.e.=0.420, $CI_{95\%}$=[-0.356;1.289], P=0.267; first exclusion rule: β=0.697, s.e.=0.406, $CI_{95\%}$=[-0.099;1.494], P=0.086; second exclusion rule: β=0.800, s.e.=0.454, $CI_{95\%}$=[-0.091;1.691], P=0.078). This suggests that the tendency to further reduce mobility on the weekends than during the week for low risk-tolerance regions (as compared to high risk-tolerance regions) is evident before pandemic declaration. Moreover, we see that the results with triple interactions between risk preference, weekend, and pandemic declaration resembles to that in the main text, albeit for regions with very high risk preference, the pre- and post-declaration difference in the weekend reduction in mobility is less precisely estimated in the second sample restriction, in particular for retail and recreation, grocery and pharmacy, and parks.

Lastly, we found some of the estimates of the risk preference-risk pool interaction terms is similar to that in the main analysis. For retail & recreation, the first exclusion rule (β=-0.365, s.e.=0.185, $CI_{95\%}$=[-0.729;-0.002], P=0.049) and second exclusion rule (β=-0.550, s.e.=0.176, $CI_{95\%}$=[-0.894;-0.205], P=0.002) both result in significant interaction terms, while transforming the government response indicators, the significance disappeared (β=-0.046, s.e.=0.163, $CI_{95\%}$=[-0.365;0.273], P=0.777). For residential area, the negative interaction terms is highly robust (first exclusion rule: β=-0.167, s.e.=0.060, $CI_{95\%}$=[-0.284;-0.050], P=0.005; second exclusion rule: β=-0.189, s.e.=0.063, $CI_{95\%}$=[-0.313;-0.066], P=0.003; government response indicators transformed: β=-0.268, s.e.=0.045, $CI_{95\%}$=[-0.355;-0.181], P<0.001). For other localities, the coefficient of the interaction term is consistently not statistically significant.

Supplementary Figures

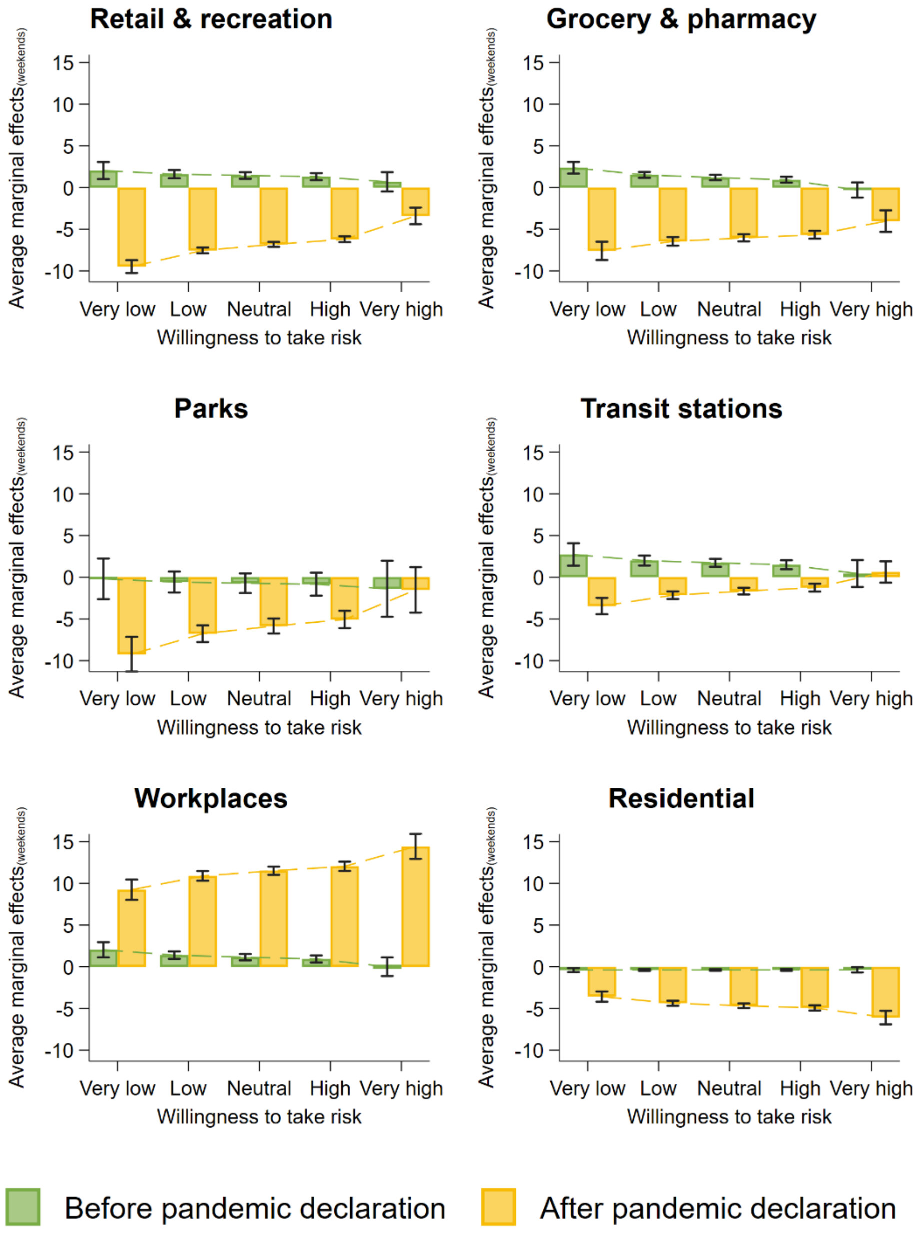

Fig. S1 | Average marginal effects of weekends on mobility changes over risk attitudes, before and after pandemic declaration.

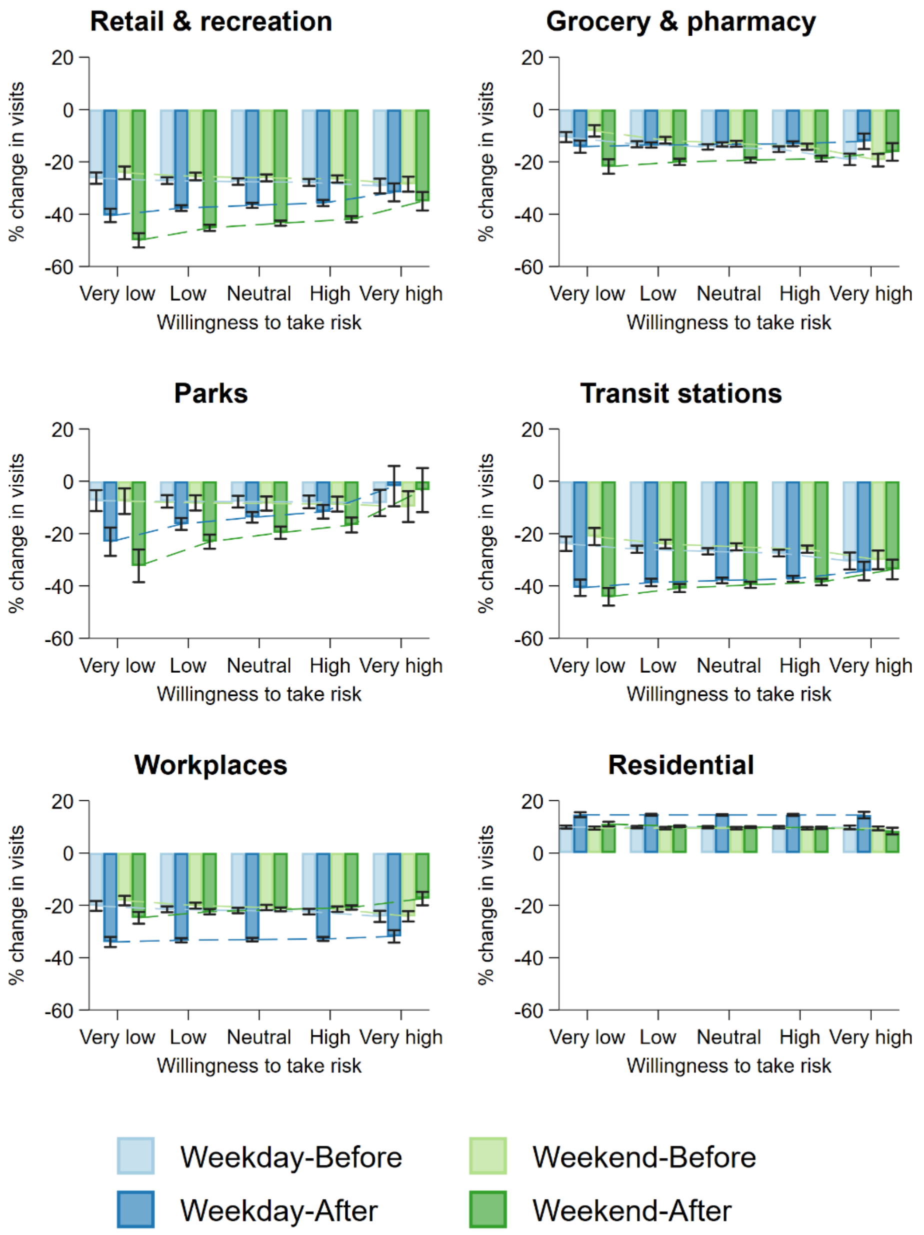

Fig. S2 | Predicted change in mobility on weekdays and weekends and before and after pandemic declaration, over risk attitudes.

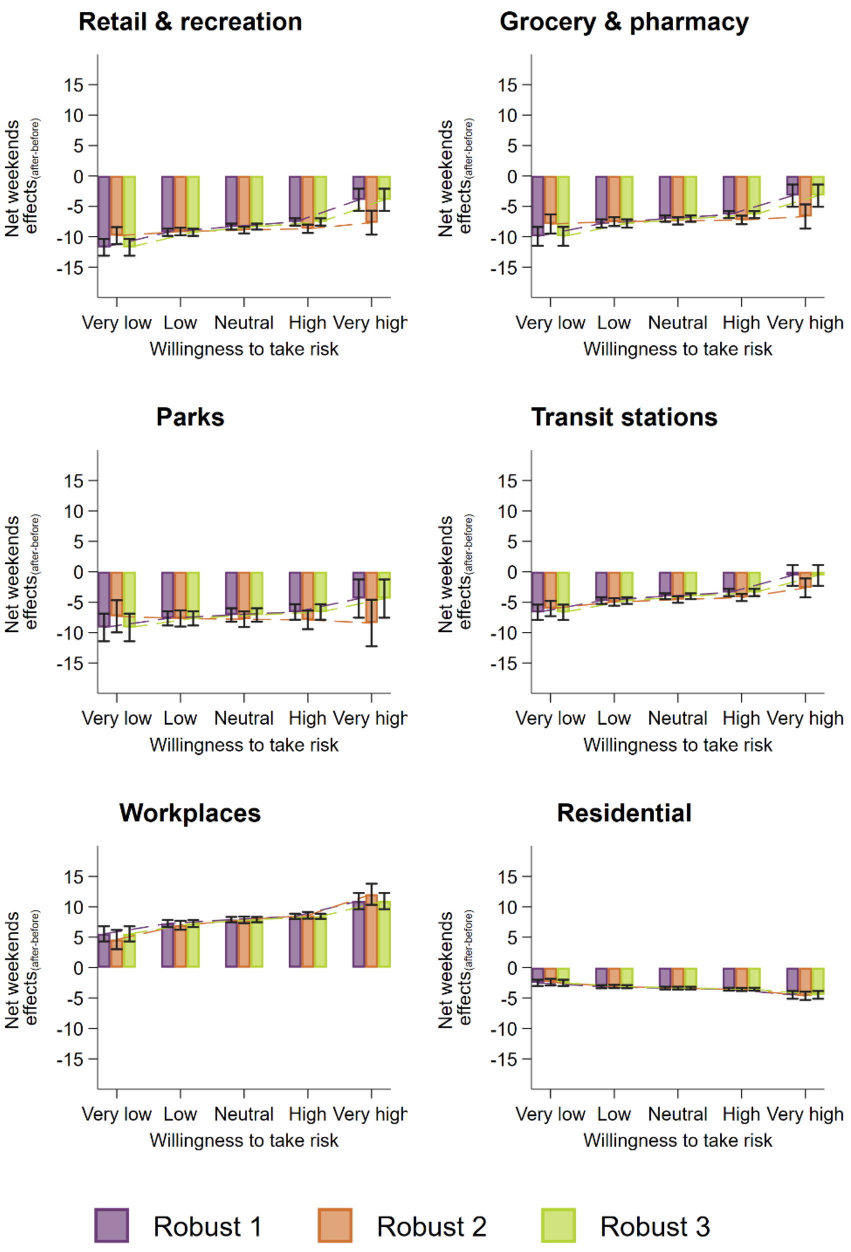

Fig. S3 | Robustness checks on mediation from risk preference to change in weekends and weekdays visiting pattern before and after pandemic declaration. Robust 1 = regions with at least one censored values on the outcome mobility measures excluded. Robust 2 = regions with at least one censored values on any mobility measures excluded. Robust 3 = government response indicators recoded as no measures taken if policy is not applied countrywide.

Supplementary Tables

Table S1 | Risk attitude and human mobility and during COVID-19

	Retail & recreation	Grocery & pharmacy	Parks	Transit stations	Workplaces	Residential
Risk-taking	2.87*	-0.48	7.67**	1.35	0.31	-0.24
	(1.180)	(1.060)	(2.577)	(1.350)	(0.848)	(0.374)
Pandemic declaration	-11.8***	-1.42*	-8.38***	-12.6***	-8.38***	3.62***
	(0.879)	(0.711)	(1.373)	(0.828)	(0.649)	(0.287)
Weekends	-4.39***	-3.97***	-4.54***	-0.79***	8.28***	-3.28***
	(0.132)	(0.167)	(0.446)	(0.189)	(0.215)	(0.110)
Days after first death	0.042†	0.13***	0.056	-0.12***	0.046*	0.017*
	(0.0216)	(0.0171)	(0.0352)	(0.0242)	(0.0190)	(0.00831)
ln(# confirmed cases+1)	-2.32***	-1.86***	1.69†	1.72***	-1.53***	0.13
	(0.466)	(0.395)	(0.881)	(0.481)	(0.358)	(0.165)
School						
Recommend closing	-7.61***	0.98	-30.9***	2.17	3.95***	2.50***
	(2.103)	(1.087)	(4.999)	(1.614)	(1.037)	(0.582)
Require closing (some)	6.68***	1.08	15.6***	5.18†	0.11	-1.43***
	(1.832)	(1.670)	(3.185)	(2.728)	(1.242)	(0.284)
Require closing	-6.65***	-5.53***	-4.26***	-2.75***	-5.92***	2.49***
	(0.764)	(0.600)	(1.151)	(0.736)	(0.598)	(0.233)
Workplace closing						
Recommend closing	-1.69†	5.47***	8.02***	-3.09***	0.90	-0.44
	(0.907)	(0.904)	(1.346)	(0.930)	(0.845)	(0.302)
Require closing (some)	-23.5***	-7.33***	-3.29†	-16.0***	-11.9***	4.03***
	(1.224)	(1.097)	(1.825)	(1.291)	(0.881)	(0.371)
Require closing	-16.8***	-4.50***	-5.76*	-17.5***	-9.79***	4.38***
	(1.334)	(1.183)	(2.416)	(1.367)	(0.956)	(0.432)
Public events						
Recommend cancelling	-0.69	2.56*	-5.61***	-4.37**	-2.34**	1.87***
	(1.180)	(1.017)	(1.322)	(1.504)	(0.793)	(0.233)
Require cancelling	-5.04***	-0.42	-3.91*	-3.78***	-4.06***	2.45***
	(0.716)	(0.687)	(1.652)	(0.869)	(0.674)	(0.229)
Restrictions on gatherings						
Above 1000 people	10.5***	8.94***	1.69	4.17***	6.68***	-3.87***
	(1.003)	(1.091)	(2.010)	(1.258)	(0.883)	(0.320)
101-1000 people	4.97***	8.12***	2.58	2.30†	5.58***	-2.41***
	(1.245)	(1.077)	(2.041)	(1.366)	(0.861)	(0.323)
11-100 people	-1.29	-1.06	-5.11*	-3.58*	-4.64***	0.011
	(1.398)	(1.245)	(2.456)	(1.504)	(1.083)	(0.430)
10 people or less	-1.68	-0.59	-3.62†	-3.53**	-2.67**	-0.21
	(1.192)	(1.065)	(1.877)	(1.257)	(0.915)	(0.357)
Public transport						
Recommend closing	-8.52***	-9.87***	-10.4***	-8.77***	-10.8***	4.31***
	(0.795)	(0.702)	(1.559)	(0.770)	(0.613)	(0.253)
Require closing	-7.44***	-11.6***	-18.3***	-6.73***	-5.22***	2.84***
	(1.368)	(1.316)	(2.194)	(1.299)	(1.003)	(0.473)
Stay at home requirements						
Recommend not leaving house	3.40**	0.93	3.91*	1.89†	3.57***	-0.93**
	(1.172)	(0.815)	(1.566)	(1.071)	(0.723)	(0.314)
Require not leaving (loose)	-6.00***	-6.71***	-5.35*	-4.59***	-5.04***	2.98***
	(1.315)	(1.124)	(2.227)	(1.201)	(0.883)	(0.373)
Require not leaving (strict)	-19.5***	-25.7***	-18.2***	-15.0***	-13.0***	9.68***
	(1.757)	(1.526)	(3.059)	(1.809)	(1.349)	(0.569)
Internal movement						
Recommend movement restriction	-9.05***	-6.87***	-7.09***	-10.4***	-3.70***	1.98***
	(0.841)	(0.665)	(1.714)	(0.828)	(0.530)	(0.258)
Restrict movement	-7.75***	-6.59***	-1.99	-8.96***	-6.41***	1.97***
	(1.180)	(0.836)	(2.681)	(1.086)	(0.702)	(0.355)
% population ages 65	-0.023	-0.71***	-0.35	-0.30†	-0.36***	-0.032

and above	(0.135)	(0.141)	(0.396)	(0.166)	(0.0924)	(0.0411)
Population density (per sq. km)	-0.013**	-0.0014	-0.0048	-0.0051	-0.010**	0.0049***
	(0.00419)	(0.00379)	(0.0108)	(0.00590)	(0.00357)	(0.00119)
Unemployment (% labour force)	-0.84***	-0.37***	-1.70***	-0.66***	-0.27***	0.21***
	(0.0978)	(0.100)	(0.250)	(0.0995)	(0.0639)	(0.0320)
GDP per capita (2010 US$ constant)	1.74†	6.72***	19.7***	3.97**	-0.57	-1.11**
	(1.057)	(1.018)	(3.100)	(1.250)	(0.789)	(0.413)
Urban population (% total)	-0.18***	-0.25***	-0.99***	-0.34***	-0.10**	0.078***
	(0.0382)	(0.0413)	(0.102)	(0.0531)	(0.0336)	(0.0144)
Average temperature (tenths of °C)	0.030***	0.025***	0.19***	0.037***	-0.0019	-0.015***
	(0.00436)	(0.00402)	(0.0103)	(0.00475)	(0.00336)	(0.00138)
Average household size	-1.27	-3.60*	-12.7*	-2.22	0.74	0.27
	(1.075)	(1.506)	(5.192)	(1.485)	(0.771)	(0.449)
Constant	8.05	-23.3*	-81.8*	0.73	19.5**	5.31
	(10.31)	(11.73)	(38.70)	(12.70)	(7.033)	(4.377)
Observations	64800	64613	58858	62958	67073	58284
Number of clusters	796	785	738	761	798	741
Prob. > χ^2	0.000	0.000	0.000	0.000	0.000	0.000
$R^2_{between}$	0.849	0.578	0.367	0.821	0.782	0.823
R^2_{within}	0.519	0.427	0.193	0.217	0.512	0.557
$R^2_{overall}$	0.789	0.538	0.290	0.700	0.743	0.771

Notes: Results corresponds to Figure 1 in the main text. Random-effects GLS regression estimates. Standard errors (clustered at regional level) in parentheses. † $p < .10$; * $p < .05$; ** $p < .01$; *** $p < .001$. Reference categories are: *Before WHO declares COVID-19 as pandemic, Weekdays* and *No measures taken* for all government response indicators.

Table S2 | Change in visits to six location categories predicted by average individual risk preference before and after pandemic declaration.

	Retail & recreation	Grocery & pharmacy	Parks	Transit stations	Workplaces	Residential
Risk-taking	-1.55	-4.45***	-0.46	-3.53*	-2.40**	-0.067
	(1.078)	(0.898)	(1.963)	(1.382)	(0.797)	(0.234)
Pandemic declaration	-11.3***	-0.99	-7.30***	-12.0***	-8.10***	3.60***
	(0.879)	(0.705)	(1.379)	(0.833)	(0.642)	(0.285)
Pandemic declaration	6.72***	5.98***	11.9***	7.17***	4.02***	-0.27
*Risk-taking	(1.166)	(1.013)	(2.449)	(1.422)	(0.871)	(0.423)
Weekends	-4.40***	-3.98***	-4.55***	-0.80***	8.27***	-3.28***
	(0.132)	(0.167)	(0.447)	(0.189)	(0.215)	(0.110)
Days after first death	0.047*	0.14***	0.062†	-0.11***	0.048**	0.017*
	(0.0211)	(0.0167)	(0.0350)	(0.0236)	(0.0187)	(0.00831)
ln(# confirmed cases+1)	-2.41***	-1.94***	1.52†	1.60***	-1.58***	0.14
	(0.461)	(0.390)	(0.871)	(0.471)	(0.354)	(0.164)
School						
Recommend closing	-7.23***	1.30	-30.6***	2.46	4.20***	2.49***
	(2.142)	(1.099)	(5.076)	(1.632)	(1.047)	(0.583)
Require closing (some)	6.78***	1.18	15.7***	5.37*	0.18	-1.44***
	(1.845)	(1.677)	(3.227)	(2.731)	(1.248)	(0.287)
Require closing	-6.48***	-5.37***	-3.95***	-2.55***	-5.82***	2.48***
	(0.796)	(0.603)	(1.164)	(0.747)	(0.611)	(0.235)
Workplace closing						
Recommend closing	-1.69†	5.49***	8.33***	-2.95**	0.93	-0.44
	(0.881)	(0.886)	(1.351)	(0.910)	(0.832)	(0.302)
Require closing (some)	-23.1***	-6.99***	-2.45	-15.5***	-11.6***	4.01***
	(1.206)	(1.091)	(1.817)	(1.273)	(0.876)	(0.369)
Require closing	-16.4***	-4.17***	-5.07*	-17.1***	-9.51***	4.37***
	(1.316)	(1.182)	(2.403)	(1.345)	(0.951)	(0.433)
Public events						
Recommend cancelling	-0.85	2.40*	-5.91***	-4.61**	-2.45**	1.88***
	(1.172)	(1.009)	(1.313)	(1.496)	(0.798)	(0.235)
Require cancelling	-5.51***	-0.84	-4.83**	-4.32***	-4.36***	2.47***
	(0.711)	(0.686)	(1.637)	(0.863)	(0.678)	(0.230)
Restrictions on gatherings						
Above 1000 people	11.1***	9.52***	2.61	4.90***	7.09***	-3.89***
	(1.015)	(1.093)	(2.042)	(1.264)	(0.888)	(0.320)
101-1000 people	4.70***	7.89***	1.92	1.96	5.42***	-2.39***
	(1.263)	(1.086)	(2.100)	(1.380)	(0.875)	(0.327)
11-100 people	-2.10	-1.75	-6.58**	-4.46**	-5.11***	0.049
	(1.418)	(1.267)	(2.381)	(1.535)	(1.103)	(0.444)
10 people or less	-2.12†	-0.95	-4.27*	-4.00**	-2.92**	-0.19
	(1.213)	(1.083)	(1.923)	(1.277)	(0.929)	(0.363)
Public transport						
Recommend closing	-8.43***	-9.79***	-10.1***	-8.61***	-10.8***	4.30***
	(0.792)	(0.696)	(1.558)	(0.772)	(0.613)	(0.254)
Require closing	-7.14***	-11.3***	-17.5***	-6.31***	-5.13***	2.82***
	(1.359)	(1.317)	(2.194)	(1.295)	(1.000)	(0.475)
Stay at home requirements						
Recommend not leaving	3.46**	0.96	4.06**	1.96†	3.58***	-0.94**
house	(1.180)	(0.831)	(1.571)	(1.097)	(0.744)	(0.317)
Require not leaving	-6.22***	-6.93***	-5.55*	-4.76***	-5.17***	2.98***
(loose)	(1.310)	(1.128)	(2.221)	(1.209)	(0.890)	(0.372)
Require not leaving	-19.7***	-25.9***	-18.6***	-15.3***	-13.2***	9.68***
(strict)	(1.720)	(1.530)	(3.022)	(1.783)	(1.329)	(0.570)
Internal movement						
Recommend movement	-8.38***	-6.28***	-6.26***	-9.79***	-3.33***	1.96***
restriction	(0.843)	(0.675)	(1.724)	(0.836)	(0.533)	(0.255)
Restrict movement	-7.68***	-6.53***	-2.36	-9.04***	-6.41***	1.98***
	(1.157)	(0.823)	(2.654)	(1.065)	(0.696)	(0.354)
% population ages 65	-0.067	-0.75***	-0.44	-0.35*	-0.38***	-0.030

and above	(0.135)	(0.143)	(0.402)	(0.167)	(0.0915)	(0.0413)
Population density (per sq. km)	-0.013**	-0.0017	-0.0056	-0.0055	-0.011**	0.0049***
	(0.00421)	(0.00381)	(0.0108)	(0.00591)	(0.00357)	(0.00120)
Unemployment (% labour force)	-0.85***	-0.38***	-1.71***	-0.67***	-0.28***	0.21***
	(0.0981)	(0.102)	(0.251)	(0.101)	(0.0644)	(0.0321)
GDP per capita (2010 US$ constant)	1.96†	6.91***	20.2***	4.28***	-0.45	-1.12**
	(1.065)	(1.028)	(3.129)	(1.260)	(0.787)	(0.412)
Urban population (% total)	-0.19***	-0.25***	-1.00***	-0.35***	-0.11**	0.079***
	(0.0386)	(0.0415)	(0.102)	(0.0533)	(0.0336)	(0.0144)
Average temperature (tenths of °C)	0.032***	0.027***	0.19***	0.039***	-0.00050	-0.015***
	(0.00428)	(0.00401)	(0.0103)	(0.00468)	(0.00332)	(0.00137)
Average household size	-1.50	-3.79*	-13.1*	-2.42	0.59	0.28
	(1.098)	(1.541)	(5.299)	(1.517)	(0.760)	(0.450)
Constant	7.47	-23.9*	-84.7*	-0.93	19.2**	5.37
	(10.44)	(11.94)	(39.29)	(12.88)	(7.014)	(4.379)
Observations	64800	64613	58858	62958	67073	58284
Number of clusters	796	785	738	761	798	741
Prob. > χ^2	0.000	0.000	0.000	0.000	0.000	0.000
$R^2_{between}$	0.850	0.581	0.372	0.823	0.783	0.823
R^2_{within}	0.520	0.426	0.191	0.218	0.512	0.557
$R^2_{overall}$	0.791	0.540	0.291	0.702	0.744	0.771

Notes: Results corresponds to Figure 2 in the main text. Random-effects GLS regression estimates. Standard errors (clustered at regional level) in parentheses. † $p < .10$; * $p < .05$; ** $p < .01$; *** $p < .001$. Reference categories are: *Before WHO declares COVID-19 as pandemic, Weekdays* and *No measures taken* for all government response indicators.

Table S3 | Visitation pattern by weekdays and weekends over average individual risk preference.

	Retail & recreation	Grocery & pharmacy	Parks	Transit stations	Workplaces	Residential
Risk-taking	2.33*	-0.73	7.00**	1.04	-0.064	-0.057
	(1.184)	(1.050)	(2.537)	(1.352)	(0.862)	(0.391)
Weekends	-4.27***	-3.92***	-4.39***	-0.72***	8.34***	-3.35***
	(0.130)	(0.164)	(0.450)	(0.185)	(0.213)	(0.109)
Weekends*Risk-taking	2.01***	0.92*	2.26*	1.18*	1.38**	-0.79**
	(0.318)	(0.411)	(1.015)	(0.502)	(0.506)	(0.260)
Pandemic declaration	-11.8***	-1.42*	-8.38***	-12.5***	-8.38***	3.62***
	(0.879)	(0.711)	(1.373)	(0.828)	(0.648)	(0.287)
Days after first death	0.042†	0.13***	0.056	-0.12***	0.046*	0.017*
	(0.0216)	(0.0171)	(0.0351)	(0.0242)	(0.0190)	(0.00831)
ln(# confirmed cases+1)	-2.31***	-1.86***	1.70†	1.72***	-1.52***	0.13
	(0.466)	(0.395)	(0.881)	(0.482)	(0.358)	(0.165)
School						
Recommend closing	-7.77***	0.90	-31.1***	2.07	3.84***	2.57***
	(2.111)	(1.086)	(5.010)	(1.617)	(1.033)	(0.585)
Require closing (some)	6.62***	1.05	15.5***	5.15†	0.066	-1.41***
	(1.834)	(1.670)	(3.189)	(2.729)	(1.246)	(0.285)
Require closing	-6.68***	-5.54***	-4.29***	-2.77***	-5.94***	2.50***
	(0.763)	(0.599)	(1.151)	(0.737)	(0.598)	(0.233)
Workplace closing						
Recommend closing	-1.69†	5.48***	8.02***	-3.09***	0.90	-0.44
	(0.907)	(0.904)	(1.346)	(0.930)	(0.845)	(0.302)
Require closing (some)	-23.5***	-7.33***	-3.30†	-16.0***	-11.9***	4.03***
	(1.224)	(1.097)	(1.825)	(1.290)	(0.881)	(0.371)
Require closing	-16.8***	-4.49***	-5.76*	-17.5***	-9.78***	4.38***
	(1.334)	(1.183)	(2.416)	(1.367)	(0.956)	(0.432)
Public events						
Recommend cancelling	-0.64	2.58*	-5.57***	-4.34**	-2.31**	1.86***
	(1.180)	(1.017)	(1.323)	(1.503)	(0.791)	(0.233)
Require cancelling	-5.06***	-0.42	-3.91*	-3.79***	-4.06***	2.45***
	(0.717)	(0.687)	(1.652)	(0.869)	(0.675)	(0.229)
Restrictions on gatherings						
Above 1000 people	10.5***	8.96***	1.72	4.18***	6.69***	-3.87***
	(1.003)	(1.091)	(2.009)	(1.258)	(0.883)	(0.320)
101-1000 people	5.04***	8.15***	2.64	2.34†	5.63***	-2.43***
	(1.246)	(1.078)	(2.041)	(1.367)	(0.862)	(0.323)
11-100 people	-1.23	-1.03	-5.06*	-3.54*	-4.60***	-0.0035
	(1.397)	(1.245)	(2.454)	(1.505)	(1.083)	(0.430)
10 people or less	-1.65	-0.58	-3.59†	-3.51**	-2.65**	-0.21
	(1.191)	(1.065)	(1.877)	(1.257)	(0.914)	(0.357)
Public transport						
Recommend closing	-8.53***	-9.88***	-10.4***	-8.78***	-10.8***	4.31***
	(0.795)	(0.702)	(1.560)	(0.770)	(0.613)	(0.253)
Require closing	-7.46***	-11.6***	-18.3***	-6.75***	-5.24***	2.84***
	(1.367)	(1.317)	(2.194)	(1.298)	(1.003)	(0.473)
Stay at home requirements						
Recommend not leaving house	3.41**	0.94	3.92*	1.90†	3.58***	-0.94**
	(1.172)	(0.815)	(1.565)	(1.071)	(0.722)	(0.314)
Require not leaving (loose)	-5.98***	-6.70***	-5.33*	-4.58***	-5.03***	2.97***
	(1.315)	(1.123)	(2.227)	(1.201)	(0.881)	(0.373)
Require not leaving (strict)	-19.5***	-25.7***	-18.2***	-15.0***	-12.9***	9.67***
	(1.756)	(1.526)	(3.060)	(1.808)	(1.348)	(0.569)
Internal movement						
Recommend movement restriction	-9.10***	-6.89***	-7.14***	-10.4***	-3.73***	2.00***
	(0.841)	(0.665)	(1.716)	(0.828)	(0.528)	(0.258)
Restrict movement	-7.76***	-6.59***	-1.99	-8.96***	-6.41***	1.97***
	(1.180)	(0.835)	(2.681)	(1.085)	(0.701)	(0.355)
% population ages 65 and above	-0.019	-0.71***	-0.35	-0.30†	-0.36***	-0.033
	(0.135)	(0.142)	(0.396)	(0.166)	(0.0926)	(0.0412)

Population density (per sq. km)	-0.013**	-0.0014	-0.0049	-0.0051	-0.010**	0.0049***
	(0.00420)	(0.00380)	(0.0108)	(0.00590)	(0.00357)	(0.00119)
Unemployment (% labour force)	-0.84***	-0.37***	-1.70***	-0.66***	-0.27***	0.21***
	(0.0976)	(0.100)	(0.249)	(0.0994)	(0.0639)	(0.0320)
GDP per capita (2010 US$ constant)	1.72	6.71***	19.6***	3.96**	-0.57	-1.10**
	(1.056)	(1.018)	(3.101)	(1.251)	(0.790)	(0.413)
Urban population (% total)	-0.18***	-0.25***	-0.99***	-0.34***	-0.10**	0.078***
	(0.0382)	(0.0413)	(0.102)	(0.0531)	(0.0336)	(0.0144)
Average temperature (tenths of °C)	0.030***	0.025***	0.19***	0.037***	-0.0020	-0.015***
	(0.00436)	(0.00402)	(0.0103)	(0.00475)	(0.00336)	(0.00139)
Average household size	-1.26	-3.59*	-12.7*	-2.22	0.74	0.27
	(1.075)	(1.506)	(5.197)	(1.486)	(0.770)	(0.449)
Constant	8.12	-23.3*	-81.7*	0.78	19.5**	5.29
	(10.30)	(11.73)	(38.73)	(12.71)	(7.036)	(4.379)
Observations	64800	64613	58858	62958	67073	58284
Number of clusters	796	785	738	761	798	741
Prob. > χ^2	0.000	0.000	0.000	0.000	0.000	0.000
R^2_{between}	0.849	0.579	0.367	0.822	0.782	0.823
R^2_{within}	0.520	0.427	0.193	0.216	0.511	0.557
R^2_{overall}	0.790	0.538	0.291	0.700	0.743	0.771

Notes: Results corresponds to Figure 3 in the main text. Random-effects GLS regression estimates. Standard errors (clustered at regional level) in parentheses. † $p < .10$; * $p < .05$; ** $p < .01$; *** $p < .001$. Reference categories are: *Before WHO declares COVID-19 as pandemic, Weekdays* and *No measures taken* for all government response indicators.

Table S4 | Mediation from risk preference to change in weekends and weekdays visiting pattern pre- and post-pandemic declaration.

	Retail & recreation	Grocery & pharmacy	Parks	Transit stations	Workplaces	Residential
Risk-taking	-1.22	-3.93***	-0.11	-3.01*	-1.94*	-0.072
	(1.079)	(0.879)	(1.913)	(1.360)	(0.848)	(0.242)
Weekends	1.51***	1.22***	-1.16†	1.87***	1.20***	-0.43***
	(0.205)	(0.164)	(0.623)	(0.245)	(0.192)	(0.0542)
Weekends*Risk-taking	-1.32*	-1.88***	-1.48	-1.88*	-1.28**	0.10
	(0.552)	(0.399)	(1.387)	(0.762)	(0.469)	(0.152)
Pandemic declaration	-8.93***	1.11†	-5.97***	-11.0***	-11.0***	4.71***
	(0.861)	(0.675)	(1.336)	(0.813)	(0.634)	(0.285)
Pandemic declaration *Risk-taking	5.46***	4.87***	10.3***	5.94***	2.80**	0.057
	(1.131)	(0.958)	(2.346)	(1.359)	(0.886)	(0.443)
Weekends*Pandemic declaration	-8.52***	-7.54***	-4.67***	-3.75***	10.4***	-4.36***
	(0.243)	(0.261)	(0.582)	(0.266)	(0.249)	(0.124)
Weekends*Pandemic declaration*Risk-taking	5.04***	4.27***	5.99***	4.70***	4.01***	-1.40***
	(0.707)	(0.698)	(1.532)	(0.884)	(0.665)	(0.290)
Days after first death	0.049*	0.14***	0.063†	-0.11***	0.045*	0.019*
	(0.0211)	(0.0166)	(0.0349)	(0.0236)	(0.0188)	(0.00833)
ln(# confirmed cases+1)	-2.38***	-1.92***	1.55†	1.62***	-1.58***	0.12
	(0.459)	(0.388)	(0.871)	(0.470)	(0.356)	(0.165)
School						
Recommend closing	-6.88**	1.63	-30.5***	2.53	3.25**	2.92***
	(2.129)	(1.105)	(5.095)	(1.632)	(1.040)	(0.604)
Require closing (some)	6.85***	1.25	15.9***	5.39*	0.046	-1.40***
	(1.849)	(1.689)	(3.232)	(2.735)	(1.242)	(0.284)
Require closing	-6.49***	-5.39***	-3.95***	-2.54***	-5.80***	2.50***
	(0.807)	(0.612)	(1.169)	(0.751)	(0.598)	(0.230)
Workplace closing						
Recommend closing	-1.67†	5.51***	8.36***	-2.94**	0.98	-0.46
	(0.885)	(0.893)	(1.353)	(0.913)	(0.818)	(0.299)
Require closing (some)	-23.1***	-7.03***	-2.43	-15.5***	-11.6***	3.94***
	(1.206)	(1.090)	(1.817)	(1.272)	(0.876)	(0.370)
Require closing	-16.5***	-4.21***	-5.07*	-17.1***	-9.49***	4.35***
	(1.314)	(1.182)	(2.404)	(1.344)	(0.954)	(0.434)
Public events						
Recommend cancelling	-1.02	2.23*	-5.98***	-4.69**	-2.15**	1.70***
	(1.170)	(1.006)	(1.319)	(1.494)	(0.794)	(0.232)
Require cancelling	-4.93***	-0.31	-4.58**	-4.08***	-5.09***	2.74***
	(0.708)	(0.689)	(1.632)	(0.860)	(0.687)	(0.237)
Restrictions on gatherings						
Above 1000 people	10.7***	9.13***	2.40	4.72***	7.69***	-4.10***
	(1.014)	(1.094)	(2.043)	(1.264)	(0.893)	(0.323)
101-1000 people	4.20***	7.43***	1.75	1.74	6.13***	-2.64***
	(1.266)	(1.095)	(2.106)	(1.384)	(0.870)	(0.325)
11-100 people	-2.82*	-2.39†	-6.97**	-4.79**	-4.18***	-0.26
	(1.418)	(1.272)	(2.390)	(1.534)	(1.109)	(0.449)
10 people or less	-2.67*	-1.44	-4.56*	-4.26***	-2.22*	-0.47
	(1.220)	(1.087)	(1.926)	(1.279)	(0.924)	(0.364)
Public transport						
Recommend closing	-8.47***	-9.83***	-10.1***	-8.65***	-10.8***	4.27***
	(0.789)	(0.696)	(1.561)	(0.771)	(0.616)	(0.256)
Require closing	-7.22***	-11.4***	-17.6***	-6.36***	-5.14***	2.81***
	(1.352)	(1.314)	(2.196)	(1.291)	(1.007)	(0.481)
Stay at home requirements						
Recommend not leaving house	3.78**	1.23	4.31**	2.15*	3.36***	-0.79*
	(1.189)	(0.835)	(1.569)	(1.096)	(0.735)	(0.315)
Require not leaving (loose)	-6.07***	-6.81***	-5.40*	-4.65***	-5.21***	3.03***
	(1.311)	(1.127)	(2.223)	(1.207)	(0.886)	(0.373)

Require not leaving (strict)	-19.5***	-25.8***	-18.4***	-15.2***	-13.1***	9.75***
	(1.715)	(1.531)	(3.024)	(1.779)	(1.335)	(0.570)
Internal movement						
Recommend movement restriction	-8.33***	-6.23***	-6.26***	-9.78***	-3.50***	2.02***
	(0.837)	(0.674)	(1.725)	(0.833)	(0.534)	(0.256)
Restrict movement	-7.66***	-6.49***	-2.33	-9.03***	-6.45***	1.98***
	(1.152)	(0.827)	(2.662)	(1.063)	(0.699)	(0.353)
% population ages 65 and above	-0.080	-0.76***	-0.45	-0.36*	-0.37***	-0.038
	(0.135)	(0.142)	(0.402)	(0.167)	(0.0913)	(0.0417)
Population density (per sq. km)	-0.013**	-0.0016	-0.0056	-0.0055	-0.011**	0.0050***
	(0.00420)	(0.00380)	(0.0108)	(0.00590)	(0.00358)	(0.00120)
Unemployment (% labour force)	-0.85***	-0.38***	-1.71***	-0.66***	-0.28***	0.22***
	(0.0977)	(0.102)	(0.251)	(0.101)	(0.0644)	(0.0322)
GDP per capita (2010 US$ constant)	1.81†	6.80***	20.1***	4.22***	-0.29	-1.15**
	(1.062)	(1.024)	(3.126)	(1.259)	(0.785)	(0.414)
Urban population (% total)	-0.18***	-0.25***	-1.00***	-0.35***	-0.11***	0.081***
	(0.0385)	(0.0413)	(0.102)	(0.0532)	(0.0336)	(0.0145)
Average temperature (tenths of °C)	0.030***	0.025***	0.19***	0.038***	0.0020	-0.016***
	(0.00427)	(0.00398)	(0.0102)	(0.00468)	(0.00330)	(0.00137)
Average household size	-1.47	-3.76*	-13.1*	-2.41	0.54	0.29
	(1.086)	(1.527)	(5.297)	(1.515)	(0.748)	(0.459)
Constant	6.88	-24.7*	-85.1*	-1.23	20.0**	4.95
	(10.38)	(11.87)	(39.27)	(12.87)	(6.943)	(4.428)
Observations	64800	64613	58858	62958	67073	58284
Number of clusters	796	785	738	761	798	741
Prob. > χ^2	0.000	0.000	0.000	0.000	0.000	0.000
$R^2_{between}$	0.854	0.587	0.374	0.824	0.790	0.830
R^2_{within}	0.523	0.429	0.192	0.218	0.509	0.555
$R^2_{overall}$	0.794	0.545	0.293	0.703	0.750	0.777

Notes: Results corresponds to Figure 4 in the main text and Supplementary Figures S1 and S2. Random-effects GLS regression estimates. Standard errors (clustered at regional level) in parentheses. † *p* < .10; * *p* < .05; ** *p* < .01; *** *p* < .001. Reference categories are: *Before WHO declares COVID-19 as pandemic, Weekdays* and *No measures taken* for all government response indicators.

Table S5 | Change of mobility patterns based on risk preference and share of population.

	Retail & recreation	Grocery & pharmacy	Parks	Transit stations	Workplaces	Residential
Risk-taking	7.11**	-2.96	12.1**	2.67	2.10	1.82**
	(2.399)	(2.149)	(4.238)	(3.022)	(1.785)	(0.612)
% Population ages 65 and above	-0.23	-0.60***	-0.56	-0.36*	-0.45***	-0.13**
	(0.148)	(0.164)	(0.484)	(0.153)	(0.0965)	(0.0489)
Risk-taking*% Population ages 65 and above	-0.39*	0.23	-0.40	-0.12	-0.16	-0.18***
	(0.169)	(0.159)	(0.371)	(0.199)	(0.115)	(0.0498)
Weekends	-4.39***	-3.97***	-4.54***	-0.79***	8.28***	-3.28***
	(0.132)	(0.167)	(0.446)	(0.189)	(0.215)	(0.110)
Pandemic declaration	-11.8***	-1.42*	-8.39***	-12.6***	-8.38***	3.62***
	(0.879)	(0.711)	(1.374)	(0.828)	(0.649)	(0.286)
Days after first death	0.042†	0.13***	0.056	-0.12***	0.046*	0.017*
	(0.0216)	(0.0172)	(0.0351)	(0.0242)	(0.0190)	(0.00831)
ln(# confirmed cases+1)	-2.31***	-1.87***	1.70†	1.72***	-1.52***	0.14
	(0.466)	(0.395)	(0.880)	(0.481)	(0.358)	(0.165)
School						
Recommend closing	-7.61***	0.97	-30.9***	2.17	3.96***	2.50***
	(2.103)	(1.087)	(4.998)	(1.614)	(1.037)	(0.582)
Require closing (some)	6.67***	1.08	15.6***	5.18†	0.10	-1.44***
	(1.834)	(1.672)	(3.185)	(2.729)	(1.243)	(0.284)
Require closing	-6.65***	-5.53***	-4.26***	-2.75***	-5.92***	2.50***
	(0.763)	(0.599)	(1.151)	(0.736)	(0.598)	(0.234)
Workplace closing						
Recommend closing	-1.70†	5.48***	8.01***	-3.09***	0.89	-0.44
	(0.906)	(0.903)	(1.346)	(0.930)	(0.845)	(0.302)
Require closing (some)	-23.4***	-7.34***	-3.28†	-16.0***	-11.9***	4.04***
	(1.226)	(1.099)	(1.828)	(1.293)	(0.883)	(0.371)
Require closing	-16.8***	-4.50***	-5.76*	-17.5***	-9.78***	4.38***
	(1.334)	(1.183)	(2.417)	(1.368)	(0.956)	(0.432)
Public events						
Recommend cancelling	-0.71	2.58*	-5.63***	-4.37**	-2.35**	1.85***
	(1.185)	(1.023)	(1.327)	(1.508)	(0.797)	(0.233)
Require cancelling	-5.03***	-0.43	-3.89*	-3.78***	-4.05***	2.46***
	(0.715)	(0.685)	(1.652)	(0.868)	(0.674)	(0.229)
Restrictions on gatherings						
Above 1000 people	10.5***	8.95***	1.68	4.17***	6.67***	-3.87***
	(1.002)	(1.090)	(2.010)	(1.257)	(0.883)	(0.320)
101-1000 people	4.97***	8.11***	2.58	2.30†	5.58***	-2.40***
	(1.244)	(1.078)	(2.040)	(1.367)	(0.861)	(0.324)
11-100 people	-1.30	-1.05	-5.13*	-3.58*	-4.65***	0.0039
	(1.397)	(1.242)	(2.457)	(1.504)	(1.083)	(0.430)
10 people or less	-1.69	-0.58	-3.63†	-3.53**	-2.67**	-0.21
	(1.191)	(1.062)	(1.876)	(1.257)	(0.915)	(0.356)
Public transport						
Recommend closing	-8.52***	-9.87***	-10.4***	-8.77***	-10.8***	4.31***
	(0.795)	(0.701)	(1.560)	(0.770)	(0.613)	(0.253)
Require closing	-7.42***	-11.6***	-18.3***	-6.73***	-5.22***	2.85***
	(1.369)	(1.316)	(2.192)	(1.299)	(1.004)	(0.472)
Stay at home requirements						
Recommend not leaving house	3.40**	0.94	3.90*	1.89†	3.57***	-0.94**
	(1.173)	(0.815)	(1.567)	(1.072)	(0.723)	(0.314)
Require not leaving (loose)	-6.00***	-6.71***	-5.36*	-4.59***	-5.05***	2.98***
	(1.315)	(1.123)	(2.228)	(1.201)	(0.883)	(0.372)
Require not leaving (strict)	-19.5***	-25.7***	-18.2***	-15.0***	-13.0***	9.68***
	(1.756)	(1.527)	(3.058)	(1.808)	(1.349)	(0.569)
Internal movement						
Recommend movement restriction	-9.09***	-6.84***	-7.12***	-10.4***	-3.72***	1.96***
	(0.839)	(0.663)	(1.711)	(0.828)	(0.530)	(0.259)
Restrict movement	-7.77***	-6.57***	-2.00	-8.96***	-6.42***	1.96***
	(1.180)	(0.835)	(2.679)	(1.086)	(0.701)	(0.354)
Population density (per sq. km)	-0.012**	-0.0021	-0.0035	-0.0047	-0.0098**	0.0055***
	(0.00406)	(0.00362)	(0.0107)	(0.00555)	(0.00342)	(0.00117)
Unemployment (% labour	-0.89***	-0.35**	-1.75***	-0.67***	-0.29***	0.19***
	(0.0954)	(0.106)	(0.247)	(0.102)	(0.0663)	(0.0328)

force)						
GDP per capita (2010 US$ constant)	2.47*	6.31***	20.4***	4.20***	-0.25	-0.81*
	(1.092)	(1.027)	(3.114)	(1.204)	(0.780)	(0.413)
Urban population (% total)	-0.18***	-0.25***	-0.99***	-0.34***	-0.10**	0.077***
	(0.0382)	(0.0409)	(0.102)	(0.0524)	(0.0335)	(0.0142)
Average temperature (tenths of °C)	0.030***	0.025***	0.19***	0.037***	-0.0020	-0.015***
	(0.00434)	(0.00399)	(0.0103)	(0.00474)	(0.00336)	(0.00139)
Average household size	-1.69	-3.34*	-13.2*	-2.32	0.57	0.024
	(1.158)	(1.525)	(5.422)	(1.559)	(0.785)	(0.423)
Constant	4.96	-21.7†	-84.0*	-0.26	18.1**	4.59
	(10.47)	(11.44)	(38.82)	(12.52)	(6.918)	(4.184)
Observations	64800	64613	58858	62958	67073	58284
Number of clusters	796	785	738	761	798	741
Prob. > χ^2	0.000	0.000	0.000	0.000	0.000	0.000
$R^2_{between}$	0.849	0.578	0.367	0.821	0.782	0.823
R^2_{within}	0.522	0.428	0.196	0.217	0.512	0.566
$R^2_{overall}$	0.790	0.539	0.292	0.700	0.743	0.773

Notes: Results corresponds to Figure 5 in the main text. Random-effects GLS regression estimates. Standard errors (clustered at regional level) in parentheses. † $p < .10$; * $p < .05$; ** $p < .01$; *** $p < .001$. Reference categories are: *Before WHO declares COVID-19 as pandemic, Weekdays* and *No measures taken* for all government response indicators.

Table S6 | Country data availability for mobility (Google), risk preferences (GPS), and government response (OxCGRT)

Country	Google	GPS	OxCGRT	Country	Google	GPS	OxCGRT
Afghanistan	Yes	Yes	Yes	Lebanon	Yes	No	Yes
Algeria	No	Yes	Yes	Lesotho	No	No	Yes
Andorra	No	No	Yes	Libya	Yes	No	Yes
Angola	Yes	No	Yes	Liechtenstein	Yes	No	No
Antigua and Barbuda	Yes	No	No	Lithuania	Yes	Yes	No
Argentina	Yes	Yes	Yes	Luxembourg	Yes	No	Yes
Aruba	Yes	No	Yes	Madagascar	No	No	Yes
Australia	Yes	Yes	Yes	Malawi	No	Yes	Yes
Austria	Yes	Yes	Yes	Malaysia	Yes	No	Yes
Azerbaijan	No	No	Yes	Mali	Yes	No	Yes
Bahrain	Yes	No	Yes	Malta	Yes	No	No
Bangladesh	Yes	Yes	Yes	Mauritania	No	No	Yes
Barbados	Yes	No	Yes	Mauritius	Yes	No	Yes
Belarus	Yes	No	No	Mexico	Yes	Yes	Yes
Belgium	Yes	No	Yes	Moldova	Yes	Yes	Yes
Belize	Yes	No	Yes	Mongolia	Yes	No	Yes
Benin	Yes	No	No	Morocco	No	Yes	No
Bermuda	No	No	Yes	Mozambique	Yes	No	Yes
Bolivia	Yes	Yes	Yes	Myanmar (Burma)	Yes	No	Yes
Bosnia and Herzegovina	Yes	Yes	Yes	Namibia	Yes	No	Yes
Botswana	Yes	Yes	Yes	Nepal	Yes	No	No
Brazil	Yes	Yes	Yes	Netherlands	Yes	Yes	Yes
Brunei	No	No	Yes	New Zealand	Yes	No	Yes
Bulgaria	Yes	No	Yes	Nicaragua	Yes	Yes	Yes
Burkina Faso	Yes	No	Yes	Niger	Yes	No	Yes
Burundi	No	No	Yes	Nigeria	Yes	Yes	Yes
Cambodia	Yes	Yes	No	North Macedonia	Yes	No	No
Cameroon	Yes	Yes	Yes	Norway	Yes	No	Yes
Canada	Yes	Yes	Yes	Oman	Yes	No	Yes
Cape Verde	Yes	No	Yes	Pakistan	Yes	Yes	Yes
Chad	No	No	Yes	Palestine	No	No	Yes
Chile	Yes	Yes	Yes	Panama	Yes	No	Yes
China	No	Yes	Yes	Papua New Guinea	Yes	No	Yes
Colombia	Yes	Yes	Yes	Paraguay	Yes	No	Yes
Costa Rica	Yes	Yes	Yes	Peru	Yes	Yes	Yes
Croatia	Yes	Yes	Yes	Philippines	Yes	Yes	Yes
Cuba	No	No	Yes	Poland	Yes	Yes	Yes
Cyprus	No	No	Yes	Portugal	Yes	Yes	Yes
Czechia	Yes	Yes	Yes	Puerto Rico	Yes	No	Yes
Côte d'Ivoire	Yes	No	No	Qatar	Yes	No	Yes
Democratic Republic of Congo	No	No	Yes	Romania	Yes	Yes	Yes
Denmark	Yes	No	Yes	Russia	No	Yes	No

Djibouti	No	No	Yes	Rwanda	Yes	Yes	Yes
Dominican Republic	Yes	No	Yes	Réunion	Yes	No	No
Ecuador	Yes	No	Yes	San Marino	No	No	Yes
Egypt	Yes	Yes	Yes	Saudi Arabia	Yes	Yes	Yes
El Salvador	Yes	No	Yes	Senegal	Yes	No	No
Estonia	Yes	Yes	Yes	Serbia	No	Yes	No
Eswatini	No	No	Yes	Seychelles	No	No	Yes
Fiji	Yes	No	No	Sierra Leone	No	No	Yes
Finland	Yes	Yes	Yes	Singapore	Yes	No	Yes
France	Yes	Yes	Yes	Slovakia	Yes	No	Yes
Gabon	Yes	No	Yes	Slovenia	Yes	No	Yes
Gambia	No	No	Yes	South Africa	Yes	Yes	Yes
Georgia	Yes	Yes	No	South Korea	Yes	Yes	Yes
Germany	Yes	Yes	Yes	South Sudan	No	No	Yes
Ghana	Yes	Yes	Yes	Spain	Yes	Yes	Yes
Greece	Yes	Yes	Yes	Sri Lanka	Yes	Yes	Yes
Greenland	No	No	Yes	Sudan	No	No	Yes
Guam	No	No	Yes	Suriname	No	Yes	No
Guatemala	Yes	Yes	Yes	Sweden	Yes	Yes	Yes
Guinea-Bissau	Yes	No	No	Switzerland	Yes	Yes	Yes
Guyana	No	No	Yes	Syria	No	No	Yes
Haiti	Yes	Yes	No	Taiwan	Yes	No	Yes
Honduras	Yes	No	Yes	Tajikistan	Yes	No	No
Hong Kong	Yes	No	Yes	Tanzania	Yes	Yes	Yes
Hungary	Yes	Yes	Yes	Thailand	Yes	Yes	Yes
India	Yes	Yes	Yes	The Bahamas	Yes	No	No
Indonesia	Yes	Yes	Yes	Togo	Yes	No	No
Iran	No	Yes	Yes	Trinidad and Tobago	Yes	No	Yes
Iraq	Yes	Yes	Yes	Tunisia	No	No	Yes
Ireland	Yes	No	Yes	Turkey	Yes	Yes	Yes
Israel	Yes	Yes	Yes	Uganda	Yes	Yes	Yes
Italy	Yes	Yes	Yes	Ukraine	No	Yes	No
Jamaica	Yes	No	Yes	United Arab Emirates	Yes	Yes	Yes
Japan	Yes	Yes	Yes	United Kingdom	Yes	Yes	Yes
Jordan	Yes	Yes	Yes	United States	Yes	Yes	Yes
Kazakhstan	Yes	Yes	Yes	Uruguay	Yes	No	Yes
Kenya	Yes	Yes	Yes	Uzbekistan	No	No	Yes
Kosovo	No	No	Yes	Venezuela	Yes	Yes	Yes
Kuwait	Yes	No	Yes	Vietnam	Yes	Yes	Yes
Kyrgyzstan	Yes	No	Yes	Yemen	Yes	No	No
Laos	Yes	No	Yes	Zambia	Yes	No	Yes
Latvia	Yes	No	No	Zimbabwe	Yes	Yes	Yes

Note: GPS = Global Preference Survey. OxCGRT = Oxford COVID-19 Government Response Tracker. Numbers in bracket show the number of regions in the corresponding dataset.

Table S7 | Robustness checks on overall risk-mobility relationship.

**Robust 1**	Retail & recreation	Grocery & pharmacy	Parks	Transit stations	Workplaces	Residential
Risk-taking	2.22†	-0.33	8.02**	1.31	0.76	0.089
	(1.183)	(1.006)	(2.535)	(1.352)	(0.854)	(0.413)
Pandemic declaration	-12.9***	-2.48***	-10.1***	-13.0***	-8.93***	4.36***
	(0.911)	(0.744)	(1.379)	(0.822)	(0.645)	(0.323)
Weekends	-4.88***	-4.23***	-4.95***	-0.70***	8.33***	-3.39***
	(0.127)	(0.170)	(0.478)	(0.198)	(0.218)	(0.129)
% Population 65+	-0.18	-0.94***	-1.50***	-0.30†	-0.17†	0.20***
	(0.147)	(0.128)	(0.295)	(0.156)	(0.0922)	(0.0489)
Controls	Yes	Yes	Yes	Yes	Yes	Yes
Observations	58340	57571	51715	57764	63041	44546
Number of clusters	688	678	609	680	744	524
Prob. > χ^2	0.000	0.000	0.000	0.000	0.000	0.000
R^2-between	0.861	0.600	0.403	0.827	0.788	0.806
R^2-within	0.415	0.431	0.339	0.168	0.513	0.489
R^2-overall	0.798	0.558	0.371	0.704	0.750	0.761
**Robust 2**	Retail & recreation	Grocery & pharmacy	Parks	Transit stations	Workplaces	Residential
Risk-taking	2.55*	-0.28	9.46***	1.86	-0.25	0.25
	(1.074)	(0.905)	(2.589)	(1.186)	(0.773)	(0.427)
Pandemic declaration	-14.0***	-4.68***	-10.9***	-14.4***	-10.7***	4.33***
	(1.031)	(0.822)	(1.519)	(0.908)	(0.716)	(0.330)
Weekends	-4.73***	-3.84***	-4.41***	-1.42***	7.54***	-3.33***
	(0.146)	(0.208)	(0.549)	(0.235)	(0.271)	(0.134)
% Population 65+	-0.46**	-0.93***	-1.83***	-0.25†	-0.22*	0.22***
	(0.155)	(0.128)	(0.319)	(0.143)	(0.103)	(0.0514)
Controls	Yes	Yes	Yes	Yes	Yes	Yes
Observations	41146	41146	41146	41146	41146	41146
Number of clusters	484	484	484	484	484	484
Prob. > χ^2	0.000	0.000	0.000	0.000	0.000	0.000
R^2-between	0.849	0.570	0.391	0.840	0.771	0.807
R^2-within	0.583	0.572	0.372	0.321	0.638	0.508
R^2-overall	0.803	0.571	0.377	0.773	0.752	0.763
**Robust 3**	Retail & recreation	Grocery & pharmacy	Parks	Transit stations	Workplaces	Residential
Risk-taking	3.72**	-0.25	6.07*	1.60	0.58	-0.48
	(1.225)	(1.138)	(2.455)	(1.597)	(0.940)	(0.374)
Pandemic declaration	-17.2***	-3.59***	-10.7***	-18.2***	-13.7***	5.91***
	(0.790)	(0.518)	(1.084)	(0.825)	(0.612)	(0.264)
Weekends	-4.44***	-4.08***	-4.60***	-0.87***	8.31***	-3.29***
	(0.140)	(0.172)	(0.459)	(0.191)	(0.209)	(0.109)
% Population 65+	0.30*	-0.56***	-0.0023	0.099	-0.0078	-0.16**
	(0.146)	(0.160)	(0.396)	(0.192)	(0.102)	(0.0510)
Controls	Yes	Yes	Yes	Yes	Yes	Yes
Observations	64800	64613	58858	62958	67073	58284
Number of clusters	796	785	738	761	798	741
Prob. > χ^2	0.000	0.000	0.000	0.000	0.000	0.000
R^2-between	0.846	0.568	0.386	0.808	0.768	0.802
R^2-within	0.632	0.471	0.284	0.272	0.528	0.626
R^2-overall	0.809	0.537	0.344	0.697	0.730	0.764

Notes: **Robust 1** = regions with at least one censored values on the outcome mobility measures excluded. **Robust 2** = regions with at least one censored values on any mobility measures excluded. **Robust 3** = government response indicators recoded as no measures taken if policy is not applied countrywide. Random-effects GLS regression estimates. Standard errors (clustered at regional level) in parentheses. † $p < .10$; * $p < .05$; ** $p < .01$; *** $p < .001$. We controlled for number of confirmed cases (in logs), population density, and the set of government response indicators in each regression. Reference categories are: *Before WHO declares COVID-19 as pandemic*, *Weekdays* and *No measures taken*.

Table S8 | Robustness checks on moderation effect of pandemic declaration on risk-mobility relationship.

**Robust 1**	Retail & recreation	Grocery & pharmacy	Parks	Transit stations	Workplaces	Residential
Risk-taking	-1.13	-4.54***	0.91	-3.33*	-1.78*	0.24
	(1.016)	(0.806)	(1.885)	(1.340)	(0.794)	(0.223)
Pandemic declaration	-12.5***	-1.92**	-9.06***	-12.4***	-8.65***	4.33***
	(0.920)	(0.742)	(1.396)	(0.833)	(0.641)	(0.324)
Pandemic declaration*Risk-taking	4.96***	6.26***	10.5***	6.92***	3.77***	-0.22
	(1.210)	(1.075)	(2.477)	(1.514)	(0.880)	(0.515)
Controls	Yes	Yes	Yes	Yes	Yes	Yes
Observations	58340	57571	51715	57764	63041	44546
Number of clusters	688	678	609	680	744	524
Prob. > χ^2	0.000	0.000	0.000	0.000	0.000	0.000
R^2-between	0.862	0.603	0.407	0.828	0.789	0.806
R^2-within	0.418	0.432	0.337	0.170	0.512	0.489
R^2-overall	0.799	0.560	0.372	0.705	0.751	0.761
**Robust 2**	Retail & recreation	Grocery & pharmacy	Parks	Transit stations	Workplaces	Residential
Risk-taking	-0.65	-3.10***	1.54	-1.45	-1.27*	0.31
	(0.787)	(0.627)	(2.131)	(1.055)	(0.638)	(0.228)
Pandemic declaration	-13.4***	-4.16***	-9.42***	-13.8***	-10.5***	4.32***
	(1.053)	(0.835)	(1.564)	(0.937)	(0.729)	(0.331)
Pandemic declaration*Risk-taking	4.70***	4.15***	11.7***	4.88**	1.50	-0.092
	(1.388)	(1.190)	(2.695)	(1.698)	(0.995)	(0.548)
Controls	Yes	Yes	Yes	Yes	Yes	Yes
Observations	41146	41146	41146	41146	41146	41146
Number of clusters	484	484	484	484	484	484
Prob. > χ^2	0.000	0.000	0.000	0.000	0.000	0.000
R^2-between	0.850	0.572	0.395	0.841	0.771	0.807
R^2-within	0.587	0.574	0.371	0.326	0.639	0.508
R^2-overall	0.804	0.572	0.379	0.774	0.753	0.763
**Robust 3**	Retail & recreation	Grocery & pharmacy	Parks	Transit stations	Workplaces	Residential
Risk-taking	1.78	-2.17*	1.30	-0.38	0.39	-1.17***
	(1.096)	(0.945)	(2.028)	(1.517)	(0.823)	(0.263)
Pandemic declaration	-17.2***	-3.51***	-10.4***	-18.1***	-13.7***	5.95***
	(0.795)	(0.527)	(1.087)	(0.831)	(0.615)	(0.261)
Pandemic declaration*Risk-taking	2.98**	2.93**	7.07**	2.93*	0.28	1.07*
	(1.083)	(1.025)	(2.382)	(1.322)	(0.923)	(0.443)
Controls	Yes	Yes	Yes	Yes	Yes	Yes
Observations	64800	64613	58858	62958	67073	58284
Number of clusters	796	785	738	761	798	741
Prob. > χ^2	0.000	0.000	0.000	0.000	0.000	0.000
R^2-between	0.846	0.569	0.388	0.809	0.768	0.803
R^2-within	0.631	0.470	0.282	0.271	0.528	0.629
R^2-overall	0.809	0.537	0.344	0.697	0.730	0.765

Notes: **Robust 1** = regions with at least one censored values on the outcome mobility measures excluded. **Robust 2** = regions with at least one censored values on any mobility measures excluded. **Robust 3** = government response indicators recoded as no measures taken if policy is not applied countrywide. Random-effects GLS regression estimates. Standard errors (clustered at regional level) in parentheses. † $p < .10$; * $p < .05$; ** $p < .01$; *** $p < .001$. We controlled for weekend dummy, share of population over 65, day since first confirmed death, number of confirmed cases (in logs), population density, and the set of government response indicators in each regression. Reference categories are: *Before WHO declares COVID-19 as pandemic*, *Weekdays* and *No measures taken*.

Table S9 | Robustness checks on weekends-weekdays mobility change with mediation from risk attitude.

**Robust 1**	Retail & recreation	Grocery & pharmacy	Parks	Transit stations	Workplaces	Residential
Risk-taking	1.78	-0.51	7.10**	0.97	0.40	0.39
	(1.190)	(0.995)	(2.484)	(1.360)	(0.873)	(0.445)
Weekends	-4.76***	-4.18***	-4.69***	-0.63**	8.40***	-3.49***
	(0.125)	(0.166)	(0.487)	(0.193)	(0.216)	(0.128)
Weekends*Risk-taking	1.62***	0.70†	3.44**	1.25*	1.32*	-1.14***
	(0.308)	(0.406)	(1.092)	(0.541)	(0.511)	(0.305)
Controls	Yes	Yes	Yes	Yes	Yes	Yes
Observations	58340	57571	51715	57764	63041	44546
Number of clusters	688	678	609	680	744	524
Prob. > χ^2	0.000	0.000	0.000	0.000	0.000	0.000
R^2-between	0.861	0.600	0.403	0.827	0.788	0.807
R^2-within	0.416	0.431	0.339	0.168	0.513	0.489
R^2-overall	0.799	0.558	0.371	0.704	0.750	0.761
**Robust 2**	Retail & recreation	Grocery & pharmacy	Parks	Transit stations	Workplaces	Residential
Risk-taking	2.02†	-0.49	8.30**	1.35	-0.78	0.58
	(1.082)	(0.894)	(2.541)	(1.210)	(0.815)	(0.463)
Weekends	-4.55***	-3.77***	-4.01***	-1.25***	7.73***	-3.44***
	(0.140)	(0.208)	(0.563)	(0.236)	(0.276)	(0.133)
Weekends*Risk-taking	2.04***	0.80†	4.47***	1.96***	2.06**	-1.25***
	(0.349)	(0.454)	(1.306)	(0.585)	(0.635)	(0.321)
Controls	Yes	Yes	Yes	Yes	Yes	Yes
Observations	41146	41146	41146	41146	41146	41146
Number of clusters	484	484	484	484	484	484
Prob. > χ^2	0.000	0.000	0.000	0.000	0.000	0.000
R^2-between	0.849	0.571	0.391	0.840	0.771	0.807
R^2-within	0.583	0.572	0.373	0.322	0.638	0.508
R^2-overall	0.803	0.571	0.378	0.773	0.753	0.764
**Robust 3**	Retail & recreation	Grocery & pharmacy	Parks	Transit stations	Workplaces	Residential
Risk-taking	3.33**	-0.37	5.48*	1.36	0.28	-0.31
	(1.241)	(1.148)	(2.439)	(1.604)	(0.965)	(0.386)
Weekends	-4.36***	-4.05***	-4.47***	-0.82***	8.36***	-3.34***
	(0.139)	(0.170)	(0.464)	(0.186)	(0.206)	(0.108)
Weekends*Risk-taking	1.46***	0.47	2.00†	0.91†	1.12*	-0.72**
	(0.331)	(0.420)	(1.026)	(0.494)	(0.494)	(0.259)
Controls	Yes	Yes	Yes	Yes	Yes	Yes
Observations	64800	64613	58858	62958	67073	58284
Number of clusters	796	785	738	761	798	741
Prob. > χ^2	0.000	0.000	0.000	0.000	0.000	0.000
R^2-between	0.846	0.568	0.386	0.808	0.768	0.802
R^2-within	0.633	0.471	0.284	0.272	0.528	0.626
R^2-overall	0.809	0.537	0.344	0.697	0.730	0.765

Notes: **Robust 1** = regions with at least one censored values on the outcome mobility measures excluded. **Robust 2** = regions with at least one censored values on any mobility measures excluded. **Robust 3** = government response indicators recoded as no measures taken if policy is not applied countrywide. Random-effects GLS regression estimates. Standard errors (clustered at regional level) in parentheses. † $p < .10$; * $p < .05$; ** $p < .01$; *** $p < .001$. We controlled for pandemic declaration dummy, day since first confirmed death, share of population over 65, number of confirmed cases (in logs), population density, and the set of government response indicators in each regression. Reference categories are: *Before WHO declares COVID-19 as pandemic*, *Weekdays* and *No measures taken*.

Table S10 | Robustness checks on the moderating effect of pandemic declaration on weekends-weekdays mobility change based on risk preference.

**Robust 1**	Retail & recreation	Grocery & pharmacy	Parks	Transit stations	Workplaces	Residential
Risk-taking	-0.88	-4.05***	0.79	-2.72*	-1.24	0.16
	(1.020)	(0.777)	(1.836)	(1.316)	(0.846)	(0.229)
Weekends	1.39***	1.06***	-0.29	1.98***	1.04***	-0.38***
	(0.214)	(0.167)	(0.673)	(0.248)	(0.191)	(0.0569)
Weekends*Risk-taking	-1.06†	-1.83***	0.31	-2.18**	-1.50**	0.10
	(0.588)	(0.426)	(1.384)	(0.792)	(0.470)	(0.165)
Pandemic declaration	-10.0***	0.21	-7.31***	-11.4***	-11.7***	5.55***
	(0.905)	(0.707)	(1.338)	(0.813)	(0.631)	(0.327)
Pandemic declaration*Risk-taking	3.95***	5.27***	9.25***	5.53***	2.47**	0.31
	(1.195)	(1.011)	(2.376)	(1.448)	(0.893)	(0.550)
Weekends*Pandemic declaration	-8.93***	-7.59***	-6.37***	-3.78***	10.7***	-4.52***
	(0.242)	(0.271)	(0.588)	(0.273)	(0.248)	(0.140)
Weekends*Pandemic declaration*Risk-taking	4.12***	3.93***	5.03***	5.28***	4.25***	-1.82***
	(0.690)	(0.717)	(1.419)	(0.933)	(0.658)	(0.335)
Controls	Yes	Yes	Yes	Yes	Yes	Yes
Observations	58340	57571	51715	57764	63041	44546
Number of clusters	688	678	609	680	744	524
Prob. > χ^2	0.000	0.000	0.000	0.000	0.000	0.000
R^2-between	0.866	0.609	0.409	0.829	0.796	0.815
R^2-within	0.422	0.435	0.339	0.171	0.508	0.485
R^2-overall	0.803	0.566	0.374	0.707	0.757	0.767
**Robust 2**	Retail & recreation	Grocery & pharmacy	Parks	Transit stations	Workplaces	Residential
Risk-taking	-1.01	-3.11***	0.78	-1.36	-0.70	0.25
	(0.777)	(0.606)	(2.065)	(1.025)	(0.712)	(0.235)
Weekends	1.79***	1.67***	0.43	2.02***	0.63**	-0.41***
	(0.234)	(0.190)	(0.779)	(0.293)	(0.241)	(0.0590)
Weekends*Risk-taking	1.02†	-0.19	2.56	-0.45	-1.64**	0.061
	(0.596)	(0.458)	(1.673)	(0.800)	(0.564)	(0.175)
Pandemic declaration	-10.9***	-2.03**	-7.68***	-12.5***	-13.3***	5.50***
	(1.030)	(0.786)	(1.500)	(0.913)	(0.719)	(0.334)
Pandemic declaration*Risk-taking	4.43**	3.83***	11.0***	3.99*	-0.071	0.46
	(1.359)	(1.118)	(2.594)	(1.643)	(1.027)	(0.584)
Weekends*Pandemic declaration	-9.18***	-7.87***	-6.39***	-4.71***	10.3***	-4.40***
	(0.271)	(0.342)	(0.699)	(0.302)	(0.314)	(0.144)
Weekends*Pandemic declaration*Risk-taking	1.69*	1.63*	3.31†	3.73***	5.46***	-1.92***
	(0.723)	(0.775)	(1.700)	(0.869)	(0.799)	(0.343)
Controls	Yes	Yes	Yes	Yes	Yes	Yes
Observations	41146	41146	41146	41146	41146	41146
Number of clusters	484	484	484	484	484	484
Prob. > χ^2	0.000	0.000	0.000	0.000	0.000	0.000
R^2-between	0.854	0.578	0.398	0.842	0.778	0.814
R^2-within	0.590	0.576	0.373	0.328	0.637	0.504
R^2-overall	0.808	0.578	0.381	0.776	0.758	0.769
**Robust 3**	Retail & recreation	Grocery & pharmacy	Parks	Transit stations	Workplaces	Residential
Risk-taking	2.07†	-1.68†	1.54	0.088	0.90	-1.14***
	(1.092)	(0.942)	(1.976)	(1.503)	(0.871)	(0.260)
Weekends	1.64***	1.28***	-1.07†	2.01***	1.36***	-0.55***
	(0.205)	(0.163)	(0.616)	(0.231)	(0.190)	(0.0564)
Weekends*Risk-taking	-1.28*	-1.85***	-1.21	-1.73*	-1.39**	-0.036
	(0.546)	(0.406)	(1.380)	(0.719)	(0.460)	(0.147)
Pandemic declaration	-14.5***	-1.15*	-8.90***	-16.8***	-16.7***	7.10***
	(0.770)	(0.518)	(1.065)	(0.823)	(0.638)	(0.270)
Pandemic declaration*Risk-taking	1.94†	1.98*	5.75*	1.88	-0.75	1.26**
	(1.051)	(0.992)	(2.289)	(1.265)	(0.956)	(0.459)
Weekends*Pandemic declaration	-8.83***	-7.82***	-4.88***	-4.08***	10.2***	-4.17***
	(0.246)	(0.257)	(0.576)	(0.273)	(0.252)	(0.128)
Weekends*Pandemic declaration*Risk-taking	4.02***	3.46***	5.03***	3.97***	3.61***	-0.99**
	(0.680)	(0.648)	(1.467)	(0.836)	(0.669)	(0.309)
Controls	Yes	Yes	Yes	Yes	Yes	Yes

Observations	64800	64613	58858	62958	67073	58284
Number of clusters	796	785	738	761	798	741
Prob. > χ^2	0.000	0.000	0.000	0.000	0.000	0.000
R^2-between	0.850	0.576	0.389	0.810	0.775	0.809
R^2-within	0.633	0.470	0.283	0.272	0.529	0.631
R^2-overall	0.813	0.542	0.345	0.698	0.736	0.770

Notes: **Robust 1** = regions with at least one censored values on the outcome mobility measures excluded. **Robust 2** = regions with at least one censored values on any mobility measures excluded. **Robust 3** = government response indicators recoded as no measures taken if policy is not applied countrywide. Random-effects GLS regression estimates. Standard errors (clustered at regional level) in parentheses. † $p < .10$; * $p < .05$; ** $p < .01$; *** $p < .001$. We controlled for the day since first confirmed death, share of population over 65, number of confirmed cases (in logs), population density, and the set of government response indicators in each regression. Reference categories are: *Before WHO declares COVID-19 as pandemic*, *Weekdays* and *No measures taken*.

Table S11 | Robustness checks on risk preference and share of population at risk interaction effect on mobility.

**Robust 1**	Retail & recreation	Grocery & pharmacy	Parks	Transit stations	Workplaces	Residential
Risk-taking	6.46*	-1.77	17.2***	3.19	1.74	1.84**
	(2.593)	(2.191)	(4.404)	(3.354)	(1.891)	(0.712)
% Population 65+	-0.40*	-0.86***	-1.97***	-0.39*	-0.22*	0.100†
	(0.176)	(0.164)	(0.371)	(0.156)	(0.0999)	(0.0547)
Risk-taking*	-0.37*	0.13	-0.83*	-0.17	-0.090	-0.17**
% Population 65+	(0.185)	(0.167)	(0.363)	(0.233)	(0.123)	(0.0597)
Controls	Yes	Yes	Yes	Yes	Yes	Yes
Observations	58340	57571	51715	57764	63041	44546
Number of clusters	688	678	609	680	744	524
Prob. > χ^2	0.000	0.000	0.000	0.000	0.000	0.000
R^2-between	0.861	0.600	0.403	0.827	0.788	0.806
R^2-within	0.417	0.431	0.348	0.169	0.513	0.496
R^2-overall	0.799	0.558	0.374	0.704	0.750	0.762
**Robust 2**	Retail & recreation	Grocery & pharmacy	Parks	Transit stations	Workplaces	Residential
Risk-taking	8.32***	-1.82	14.9**	4.50*	-0.95	2.24**
	(1.922)	(1.737)	(4.687)	(2.041)	(1.279)	(0.745)
% Population 65+	-0.78***	-0.85***	-2.13***	-0.40*	-0.18	0.10†
	(0.200)	(0.174)	(0.416)	(0.185)	(0.123)	(0.0570)
Risk-taking*	-0.55**	0.15	-0.52	-0.25	0.067	-0.19**
% Population 65+	(0.176)	(0.149)	(0.416)	(0.177)	(0.106)	(0.0630)
Controls	Yes	Yes	Yes	Yes	Yes	Yes
Observations	41146	41146	41146	41146	41146	41146
Number of clusters	484	484	484	484	484	484
Prob. > χ^2	0.000	0.000	0.000	0.000	0.000	0.000
R^2-between	0.849	0.571	0.391	0.840	0.771	0.807
R^2-within	0.589	0.572	0.376	0.325	0.639	0.518
R^2-overall	0.804	0.571	0.378	0.773	0.752	0.765
**Robust 3**	Retail & recreation	Grocery & pharmacy	Parks	Transit stations	Workplaces	Residential
Risk-taking	4.23	-5.50*	6.36	-1.40	-1.28	2.53***
	(2.691)	(2.358)	(4.232)	(3.551)	(1.940)	(0.661)
% Population 65+	0.27†	-0.31†	-0.016	0.24	0.080	-0.30***
	(0.147)	(0.175)	(0.473)	(0.175)	(0.0992)	(0.0528)
Risk-taking*	-0.046	0.48**	-0.026	0.27	0.17	-0.27***
% Population 65+	(0.163)	(0.159)	(0.345)	(0.209)	(0.114)	(0.0446)
Controls	Yes	Yes	Yes	Yes	Yes	Yes
Observations	64800	64613	58858	62958	67073	58284
Number of clusters	796	785	738	761	798	741
Prob. > χ^2	0.000	0.000	0.000	0.000	0.000	0.000
R^2-between	0.846	0.568	0.386	0.808	0.768	0.802
R^2-within	0.633	0.471	0.284	0.271	0.527	0.640
R^2-overall	0.809	0.539	0.344	0.697	0.730	0.768

Notes: **Robust 1** = regions with at least one censored values on the outcome mobility measures excluded. **Robust 2** = regions with at least one censored values on any mobility measures excluded. **Robust 3** = government response indicators recoded as no measures taken if policy is not applied countrywide. Random-effects GLS regression estimates. Standard errors (clustered at regional level) in parentheses. † $p < .10$; * $p < .05$; ** $p < .01$; *** $p < .001$. We controlled for weekend dummy, pandemic declaration dummy, days since first confirmed death, number of confirmed cases (in logs), population density, and the set of government response indicators in each regression. Reference categories are: *Before WHO declares COVID-19 as pandemic*, *Weekdays* and *No measures taken*.
